# Exploring Sensory Subgroups in Typical Development and Autism Spectrum Development Using Factor Mixture Modelling

**DOI:** 10.1007/s10803-021-05256-6

**Published:** 2021-09-09

**Authors:** Patrick Dwyer, Emilio Ferrer, Clifford D. Saron, Susan M. Rivera

**Affiliations:** 1 Department of Psychology, UC Davis, Davis, USA; 2 Center for Mind and Brain, UC Davis, Davis, USA; 3 MIND Institute, UC Davis, Davis, USA

**Keywords:** Autism, Sensory processing, Heterogeneity, Factor mixture modelling, Auditory event-related potentials (ERPs), Auditory P1

## Abstract

This study uses factor mixture modelling of the Short Sensory Profile (SSP) at two time points to describe subgroups of young autistic and typically-developing children. This approach allows separate SSP subscales to influence overall SSP performance differentially across subgroups. Three subgroups were described, one including almost all typically-developing participants plus many autistic participants. SSP performance of a second, largely-autistic subgroup was predominantly shaped by a subscale indexing behaviours of low energy/weakness. Finally, the third subgroup, again largely autistic, contained participants with low (or more “atypical”) SSP scores across most subscales. In this subgroup, autistic participants exhibited large P1 amplitudes to loud sounds. Autistic participants in subgroups with more atypical SSP scores had higher anxiety and more sleep disturbances.

**Supplementary Information:**

The online version contains supplementary material available at 10.1007/s10803-021-05256-6.

## Introduction

Sensory processing in Autism Spectrum Development (ASD)^1^ has historically received little attention from clinicians and researchers, and indeed sensory processing differences were only added to the *DSM* criteria for autism in 2013 (American Psychiatric Association). However, research attention to the sensory features of autism has dramatically increased in recent years (Ben-Sasson et al., 2019), and this research emphasizes the importance of this domain in the lives of many autistic people. Furthermore, external sensory inputs play a central role in negative sensory experiences in autism (see Mostafa, 2008; Madriaga, 2010), and society’s role in exposing individuals to some of these inputs should not be ignored in favour of a sole focus on sensory processing within autistic people themselves. There are, admittedly, positive aspects to sensory processing in autism, such as experiences of sensory pleasure and enhanced performance on some perceptual tasks (Mottron, 2019). Nevertheless, sensory experiences can be a cause of distress for many autistic people (Belek, 2018). Reports of atypical sensory processing in autism have been linked to anxiety (Mazurek et al., 2013; Neil et al., 2016; Uljarević et al., 2016), sleep problems (Hohn, de Veld et al., 2019; Mazurek et al., 2019), gastrointestinal problems (Mazurek et al., 2013), adaptive functioning (Ausderau et al., 2016; Williams et al., 2018a, b), and participation in activities (Little et al., 2015). Moreover, sensory over-responsivity may divert autistic people’s attention away from social information (Green et al., 2018). Perhaps most importantly, not only have autistic sensory issues been associated with existing measures of quality of life (Lin & Huang, 2019), but they have been described as being a factor of quality of life in autism in and of themselves (McConachie et al., 2019).

### Heterogeneity of Sensory Features

However, patterns of sensory processing in autism are highly heterogeneous (Uljarević et al., 2017). Importantly, if sensory processing patterns differ across individuals, then dissimilar individuals may require different interventions or accommodations to ameliorate sensory-related challenges. In an attempt to address the obstacle posed by this sensory heterogeneity, researchers have published numerous studies using clustering and mixture model analyses to define and characterize meaningful subgroups of autistic people with different patterns of sensory symptoms. Overall, these studies suggest that there is a division between an autistic phenotype with more typical sensory features and a phenotype with larger, more global sensory differences, while results regarding other distinctions (e.g., between hypo-responsiveness and hyper-responsiveness, between sensory modalities) are more inconsistent across studies (DeBoth & Reynolds, 2017). Along with factors such as the use of different measures across studies, it seems reasonable to suppose that variability in the age of participants might have contributed to some of these ambiguities; sensory subgroups in some studies differ in chronological age (Lane et al., 2014; Liss et al., 2006; Tomchek et al., 2018). Unfortunately, most studies of sensory heterogeneity in ASD are limited by their cross-sectional nature; some autistic individuals may not be diagnosed until relatively late, potentially preventing their inclusion in younger parts of samples and confounding age differences. Relatively few longitudinal studies have explored sensory subgroups in ASD (cf. Ausderau et al., 2014, 2016; Dwyer et al., 2020a). The factor mixture modelling approach that is used in the present longitudinal study allows subgroups to vary in levels of latent variables over time.

### Dimensional and Categorical Models

Theoretically speaking, factor mixture modelling partially addresses another limitation of prior studies using clustering and mixture models to explore patterns of sensory processing in autism: their implicit assumption that patterns of heterogeneity can be adequately described through defining categorical subgroups, rather than through dimensional scores. Whereas conventional latent class and latent profile mixture models assume that observed variables are independent within each subgroup or class, factor mixture models describe patterns of covariation among variables (Lubke & Muthén, 2005). In a sense, factor mixture modelling offers some degree of synthesis between the categorical and dimensional approaches to heterogeneity (Clark et al., 2013). Factor mixture modelling describes different patterns by assigning individuals to different classes on the basis of probabilities, but individuals might still vary in their levels of the continuous latent variables onto which observed variables load.

That said, the factor mixture model may not entirely resolve the theoretical difficulties involved in subgrouping with potentially continuous data. Notably, as argued by Fushing and McAssey (2010), the question of exactly how many categorical subgroups exist in a particular dataset–that is, the question of what number of classes is “optimal”–may be ill-posed unless subgroups are well-separated and non-overlapping. Notably, such a non-overlapping structure might make these subgroups visually obvious, eliminating the need for mixture modeling. Thus, the reader should bear in mind that the classes defined in the present study may not have a discrete existence as the sole subgroups that may be validly used to describe the present dataset. There might be other subgrouping solutions, with either fewer or more classes, that could also provide meaningful information about patterns in these data. In other words, mixture model classes might be best conceptualized as a descriptive tool for illustrating dimensional heterogeneity at a given level, rather than as a tool for uncovering discrete categorical entities. The fit indices offered by mixture models (see Nylund et al., 2007) could be seen as a means of identifying particularly informative solutions, as well as of reducing researcher degrees of freedom.

### Multimodal Measurement

Another obstacle to research on heterogeneity of sensory behaviour in ASD is that posed by the improper reliance on any single type of measurement. Any specific measure indexing individual differences in sensory processing may have limitations or may converge poorly with other types of measures, and for this reason, researchers have called for multimodal investigations of sensory processing heterogeneity in ASD (Uljarević et al., 2017). For example, caregiver-report questionnaires have been criticized on the grounds that caregivers lack direct insight into the internal sensory experiences of autistic people and can therefore only report on external behaviours, which might be misleading (Grandin & Panek, 2014). This is concerning, as most studies describing sensory subgroups in autism have relied on caregiver-report questionnaires (see DeBoth & Reynolds, 2017), with exceptions including a study based on a self-report questionnaire (Elwin et al., 2017) and one based primarily on auditory event-related potentials (ERPs; Dwyer et al., 2020b).

Various studies suggest that continuous associations might exist between sensory questionnaire scores and neurophysiological responses such as ERPs and event-related fields (e.g., Aoki et al., 2019; Carter Leno et al., 2018; Donkers et al., 2015, 2019; Hudac et al., 2018). Indeed, although the subgroups found by Dwyer et al. (2020b) were defined on the basis of the strength of their ERPs, they were later compared on auditory subscales of a caregiver-report questionnaire, Short Sensory Profile (SSP), which provided multimodal evidence that relatively strong brain responses to loud sounds in ASD were linked to behavioural auditory distractibility/filtering problems. The present study sample is drawn from the same project–the Autism Phenome Project (APP), a longitudinal study at the UC Davis MIND Institute–as Dwyer et al., (2020b), but it adopts essentially the opposite approach: through factor mixture modelling, subgroups are defined on the basis of covariation between SSP subscales and latent factors, after which their ERPs can be compared. In this approach, the SSP–including non-auditory subscales–dictates the formation of subgroups, but the ERPs remain available to provide a complementary multimodal perspective.

### Present Study

The present study uses factor mixture modelling to define different subgroups of participants from the APP in terms of sensory processing features from the SSP: in different models, not only factor loadings but also both longitudinal patterns of change and patterns of covariation between time points are allowed to differ across subgroups. Following the approach taken by Little et al. (2017) and by Dwyer et al. (2020b) both autistic and typically-developing participants are included in the same mixture model. This allows results from both groups to be more clearly placed in context against one another than in studies describing subgroups in ASD alone, or in studies where non-equivalent subgroups are separately defined in ASD and TD. We made the following predictions:

1. That a large majority of typically-developing participants would be assigned, on the basis of posterior probabilities, to a single class, constituting a typical pattern of covariation between subscales and overall sensory processing as reflected in the latent factor. We expected that some, but not all, autistic participants will also be assigned to this class on the basis of their posterior probabilities.
2. That other autistic participants will be assigned to one or more classes showing a pattern of factor loadings differing from the aforementioned typical class. We expected very few typically-developing participants to be assigned to this class/these classes.
3. That autistic participants in classes with atypical patterns of factor loadings would show higher anxiety, poorer sleep, and lower levels of adaptive functioning than autistic participants in the class with typical factor loadings.
4. That autistic participants in any classes where high levels of auditory distractibility and noise distress make major contributions to overall sensory features would show stronger brain responses to loud sounds, consistent with Dwyer et al. (2020a), and that these participants would also show high levels of sensory sensitivity in other modalities.

## Methods

The study was approved by the UC Davis Institutional Review Board and all procedures were in accordance with the Declaration of Helsinki.

### Participants

Autistic participants in the present study met criteria for a pervasive developmental disorder (based on DSM-IV and Collaborative Programs of Excellence in Autism Network criteria) and passed cut-off scores on the ADOS-G (Lord et al., 2000) and, for either Social or Communication subscales, on the ADI-R (Lord et al., 1994). Further details regarding the APP and participant recruitment can be found in previous publications (e.g., Libero et al., 2016; Nordahl et al., 2011). As part of the APP, caregiver-reports of sensory behaviours on the SSP were collected at two time points an average of 2.80 years apart (range 1.15–5.31 years). APP participants were included in the present study if an SSP form was returned at either APP Time 1 (ages 2 through 4) or APP Time 3 (ages 4 through 9) with complete data (i.e., no items missing) on at least one subscale. Note that between Times 1 and 3, participants returned for a Time 2 visit, but neither SSP nor ERP data were collected at Time 2. A total of 285 participants from the APP were included in the present analysis: 190 ASD (160 male, 30 female) and 95 TD (64 male, 31 female). This included 172 autistic and 87 typically-developing participants with Time 1 SSP data, as well as 87 autistic and 55 typically-developing participants with Time 3 SSP data. Data were available at both time points from 116 participants (69 ASD, 47 TD). Further information regarding participants is provided in Table 1.

**Table 1:** Descriptive characteristics of the typically-developing and autistic participants with usable SSP data at either time point, along with statistical comparisons of groups

	Time 1					Time 3				
	ASD		TD		Cliff δ^a^ with 95% CI	ASD		TD		Cliff δ^a^ with 95% CI
	Mean (SD)	Range	Mean (SD)	Range		Mean (SD)	Range	Mean (SD)	Range	
Chronological age (months)	37.63 (6.24)	25.00–56.18	36.77 (7.32)	24.48–56.57	.12 [− .03, .27]	68.78 (11.91)	51.88–112.16	67.61 (13.64)	52.01–115.78	.13 [− .07, .32]
Cognitive ability (MSEL DQ at T1; DAS GCA at T3)	63.12 (20.64)	26.85–132.45	105.26 (11.81)	79.41–128.62	− .89*** [− .93, − .83]	89.00 (24.43)	32.00–130.00	112.80 (10.52)	85.00–146.00	− .63*** [− .76, − .46]
VABS composite Scores	75.58 (11.89)	51.00–111.00	110.12 (12.06)	82.00–137.00	− .95*** [− .98, − .92]	78.38 (15.61)	47.00–122.00	112.15 (12.05)	91.00–133.00	− .90*** [− .95, − .81]

^a^Values of δ (Cliff, 1993) are denoted with * if the corresponding Wilcoxon-Mann–Whitney rank sum test *p* value is < .05, with ** if *p* < .01, and with *** if *p* < .001, uncorrected

### Measures

#### Short Sensory Profile (SSP)

The Short Sensory Profile (SSP; McIntosh et al., 1999) is a 38-item caregiver-report questionnaire that has been widely used in research on sensory features in ASD (see, e.g., Hand et al., 2017; Lane et al., 2014; Tomchek et al., 2015; Uljarević et al., 2016). Higher scores reflect relatively “typical” sensory behaviours, whereas lower scores are indicative of more “atypical” sensory behaviours, but the SSP has a contested subscale structure. McIntosh et al. (1999) defined seven SSP subscales, while Tomchek et al. (2014) defined a total of six SSP subscales. More recently, Williams et al., (2018a, b) found both of these previous solutions had an unacceptable fit, and they defined nine subscales: Low Energy/Weakness (LEW), Taste/Smell Sensitivity (TSS), Hyperactivity/Inattention (HYI), Tactile Sensitivity (TS), Movement Sensitivity (MS), Auditory Distractibility (AD), Hypo-responsiveness to Speech (HRS), Visual Sensitivity (VS), and Noise Distress (ND). As described below, the present study uses the solution offered by Z. J. Williams and colleagues. In the present study, the SSP was collected at both Time 1 and Time 3.

#### Mullen Scales of Early Learning (MSEL)

The Mullen Scales of Early Learning (MSEL; Mullen, 1995) are a standardized assessment of cognitive ability for children under 68 months; in the APP, this measure was collected at Time 1. Four of the five MSEL subscales were administered: Visual Reception (VR), Fine Motor (FM), Expressive Language (EL), and Receptive Language (RL). A ratio full-scale developmental quotient (DQ) was calculated (as mental age/chronological age ×100), along with separate developmental quotients for the verbal (VDQ) and nonverbal (NVDQ) domains.

#### Differential Ability Scales (DAS)

The Differential Ability Scales, Second Edition (DAS-II; Elliott, 2007) are a standardized assessment of cognitive ability for children aged 2–17 years; in the APP, this measure was collected at Time 3. The standardized General Conceptual Ability (GCA) score was used as an overall index of cognitive ability in the present study. Note that discrepancies between MSEL DQ and DAS GCA at Times 1 and 3 should be interpreted with caution, as prior research indicates DAS GCA and MSEL DQ are not on the same scale; DAS GCA scores are often higher than MSEL DQ (Farmer et al., 2016).

#### Vineland Adaptive Behaviour Scales (VABS)

The Vineland Adaptive Behavior Scales, Second Edition (VABS-II; Sparrow et al., 2005) are rating scales intended to assess the adaptive functioning of individuals with developmental disabilities in their natural environments. At Times 1 and 3 in the APP the VABS was collected as a caregiver-report questionnaire. The VABS yields a standardized composite adaptive behaviour score, which was employed in the present study, as well as standardized scores for communication, daily living skills, socialization, and motor skills.

#### Childhood Behavior Checklist (CBCL)

The Childhood Behavior Checklist (CBCL; Achenbach & Rescorla, 2000) is a caregiver-report questionnaire which aims to measure problematic internalizing and externalizing behaviours. The CBCL’s DSM-oriented anxiety T-score was used to index anxiety in this study. At Time 1, this anxiety score was derived from the preschool-age version of the CBCL. At Time 3, the preschool-age CBCL was obtained from 72 autistic participants and the school-age CBCL from 15 autistic participants in the present study.

#### Children’s Sleep Habits Questionnaire (CSHQ)

The Children’s Sleep Habits Questionnaire (CSHQ; Owens et al., 2000) is a 45-item questionnaire which asks parents to report the weekly frequency of problematic sleep behaviours. Although the measure has subscales indexing bedtime resistance, sleep onset delay, sleep duration, sleep anxiety, night walking, parasomnias, sleep disordered breathing, and daytime sleepiness, the composite total sleep disturbances questionnaire was used in the present study. Complete data were available from 143 autistic participants at Time 1 and 80 at Time 3.

### EEG Task and Processing

EEG data were collected at Time 1 in the APP, when participants were aged 2 through 4 years. The EEG task and processing procedures employed in the present study have been described in greater detail elsewhere (De Meo-Monteil et al., 2019; Dwyer et al., 2020b, 2021). Briefly, while participants were seated in a caregiver’s lap watching a video of their choice, approximately ~ 1200 50 ms complex tones (combining multiple frequencies) of four intensities (50, 60, 70, and 80 dB SPL) were presented binaurally using headphones at a random interstimulus interval of 1–2 s. EEG was sampled at 1000 Hz using a 61-channel electrode cap (easycap.de). Offline in BESA 5.2 (besa.de), data were average-referenced and subjected to a low-cut filter of 0.4 Hz (12 dB/octave). Epochs (− 200 ms to + 900 ms) were extracted and bad channels, trials with extreme amplitudes, and trials with mechanical artefacts were removed. In order to eliminate putatively non-neural signals (e.g., muscle tension and eye movements), remaining trials were submitted to second-order blind source identification (SOBI; Belouchrani et al., 1997) independent components analysis using a semiautomatic artifact removal tool (SMART; see Saggar et al., 2012 for details). Trials were then averaged for each participant and condition and entered into Cartool (Brunet et al., 2011), which was used to interpolate data from excluded channels using a three-dimensional spline (Perrin et al., 1987), further filter the data (second-order Butterworth, 12 dB/octave; 40 Hz high-cut, 60 Hz notch), and apply baseline correction (using the 100 ms immediately prior to stimulus onset). In the ASD group from the present study, usable ERP data were obtained from 115 participants at Time 1.

### Confirmatory Factor Analysis (CFA)

Given the existence of multiple SSP factor solutions (McIntosh et al., 1999; Tomchek et al., 2014), of which that described by Z. J. Williams et al., (2018a, b) appeared to have the greatest precision, we chose to proceed with a confirmatory factor analysis (CFA) to determine whether the Williams et al. solution would be acceptable for use in the present study. Mplus version 8.2 (Muthén & Muthén, 1998/2017) was used to estimate separate CFA models at each time point, collapsing across groups. A robust, diagonally-weighted least squares estimator (“WLSMV”) was employed. Items were defined as ordered categorical variables. Model fit was measured using Root Mean Square Error of Approximation (RMSEA), Comparative Fit Index (CFI), Tucker-Lewis Index (TLI), and Standardized Root Mean Square Residual (SRMR). Hu and Bentler’s (1999) criteria (acceptable RMSEA < 0.06, CFI/TLI > 0.95, and SRMR < 0.08) were used to evaluate model fit.

### Factor Mixture Model (FMM)

As CFA showed acceptable fit (see results section below), we proceeded to define a factor mixture model in Mplus version 8.2 using the nine SSP subscales proposed by Z. J. Williams et al., (2018a, b) as observed variables. In the overall model, at each time point, a latent factor was defined loading on all nine SSP subscales. This overall latent factor is not intended to suggest that the SSP is unidimensional; it was defined in order to explore how different subscales contribute to the individual’s overall level of sensory behaviours and whether these contributions vary across classes of individuals. To simplify the model, factor loadings were constrained to be equal across the two time-points. Covariances between scores on each SSP subscale at Time 1 and the same subscale at Time 3 were estimated, as were covariances between the latent factors at Times 1 and 3. Variances of each latent factor were fixed to one. The mean of the latent factor at Time 1 was fixed to one, and the means of subscales were constrained to be equal across the two time-points, thereby forcing any longitudinal change in sensory processing scores to be expressed through the estimated mean of the latent factor at Time 3.

The classes defined in the factor mixture model were allowed to vary from the overall model in the loadings of each SSP subscale onto the latent factor (although these loadings were still constrained to be equal across time-points). Furthermore, in some models, classes were also allowed to vary in the covariance among the latent factors at each time-point (representing the degree to which overall Time 1 sensory features predicted Time 3 sensory features) and/or in the mean of the latent factor at Time 3 (representing overall longitudinal changes in levels of total sensory features).

Fit indices were used to evaluate and compare models (Nylund et al., 2007). These included information criteria: Akaike’s Information Criterion (AIC), Bayesian Information Criterion (BIC), and sample size-adjusted BIC (SABIC). These information criteria endeavour to maximize both the parsimony and explanatory power of models; smaller values indicate better model fit. Furthermore, statistical tests were used in an effort to determine whether model fit significantly improved with the addition of new classes; these tests were the Lo-Mendell-Rubin (LMR) test, the Vuong-LMR (VLMR) test, and the bootstrap likelihood ratio test (BLRT). Through bootstrapping, the BLRT, unlike the LMR and VLMR tests, empirically estimates the shape of the distribution of differences between models. Finally, entropy was considered; entropy measures the separation of classes, with more separated classes–reflected by higher values–suggesting more powerful models (Celeux & Soromenho, 1996).

### Comparisons of Classes

After model selection, exploratory follow-up analyses were conducted to determine whether autistic participants assigned to separate classes differed in chronological age, cognitive ability, adaptive behaviour, anxiety, and scores on each SSP subscale at Time 1 or Time 3. If participants who lacked SSP data at one time point had other relevant scores at that time point, these data were included in the analysis. Kruskal–Wallis tests were used for omnibus effects and Wilcoxon-Mann–Whitney tests with Bonferroni-Holm corrections were used to determine significance of post-hoc comparisons, which are reported using the effect size δ (Cliff, 1993) with a 95% asymmetric confidence interval from the orddom R package (Rogmann, 2013). Values of δ can range from –1 to + 1, with each extreme value representing a complete lack of overlap between the ordinal data. In contrast, Cliff’s δ values approaching 0 suggest classes are indistinguishable on a given dependent variable. *η*^2^ effect sizes are also reported for Kruskal–Wallis tests (http://www.psychometrica.de/effect_size.html).

#### ERP Analysis

**Fig. 1:**
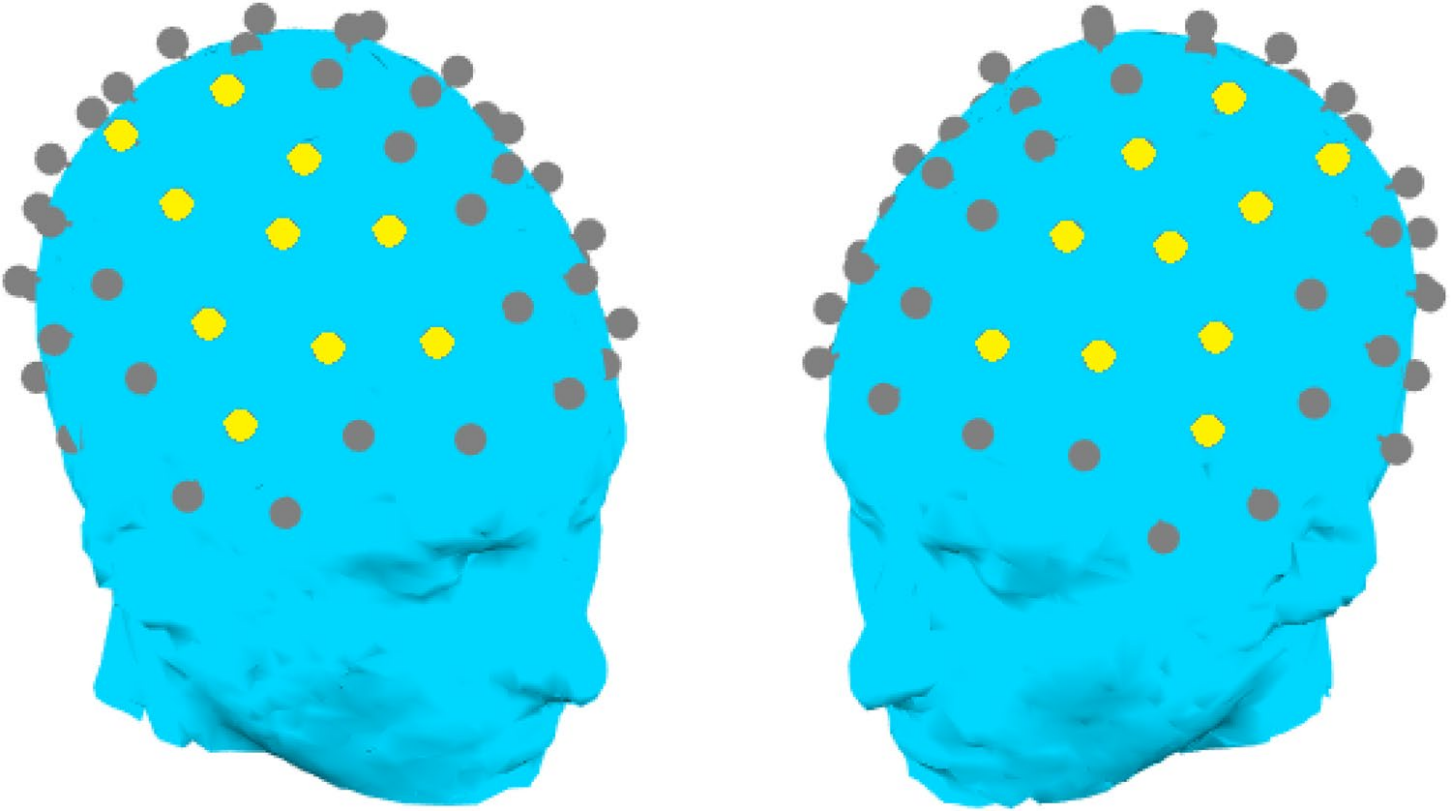
Fronto-central channels in either hemisphere selected as the measurement region for the P1 component are indicated with a yellow dot. Channel positions may appear slightly irregular; this is because channel positions are based on actual electrode positions obtained from a subset of participants using a Polhemus digitizer

ASD participants with usable ERP data at Time 1 were compared across classes using mixed ANOVA with P1 amplitude and latency as the dependent variable and stimulus intensity and hemisphere as within-subject factors. The approach used to quantify the P1 component in these ERPs has been discussed in greater detail in prior research involving the present dataset (Dwyer et al., 2021).^2^ Essentially, in order to avoid confounds with individual differences in topography or cap positioning, the P1 was measured separately in each condition and hemisphere over the fronto-central electrode at which the individual exhibited their largest positive voltage, as well as immediately adjacent electrodes; electrodes outside the pre-defined fronto-central region (see Fig. 1) were not included in the analysis. In each condition, the P1 time window was defined as the area ± 50 ms on either side the greatest positive peak in any channel in the grand-averaged data from both diagnostic groups in the full APP ERP dataset, which was 70–170 ms (50 dB), 59–159 ms (60 dB), 44–144 ms (70 dB), and 41–141 ms (80 dB). Within this time window, latency was measured as 50% fractional area latency (see Luck, 2014): that is, latency was defined as the point at which the area above and below the curve within the aforementioned time window was equalized on either side of the latency estimate. Amplitude was measured as area amplitude within the P1 time window.

## Results

### Confirmatory Factor Analysis (CFA)

At both time points, all CFA fit indices examined were within acceptable ranges, supporting the SSP factors/subscales defined by Z. J. Williams et al., (2018a, b), at least with regard to the data from autistic and typically-developing children in the present study (Table 2).

**Table 2:** Fit indices of CFA models at each time-point

Timepoint	χ^2^ (df)	RMSEA	CFI	TLI	SRMR
Time 1	613.163 (398)	.046	.985	.982	.056
Time 3	557.463 (398)	.053	.983	.980	.061

Hu and Bentler (1999) suggest that acceptable values for RMSEA are < .06, for CFI and TLI > .95, and for SRMR < .08

### Fit Indices and Selection of FMM

**Fig. 2:**
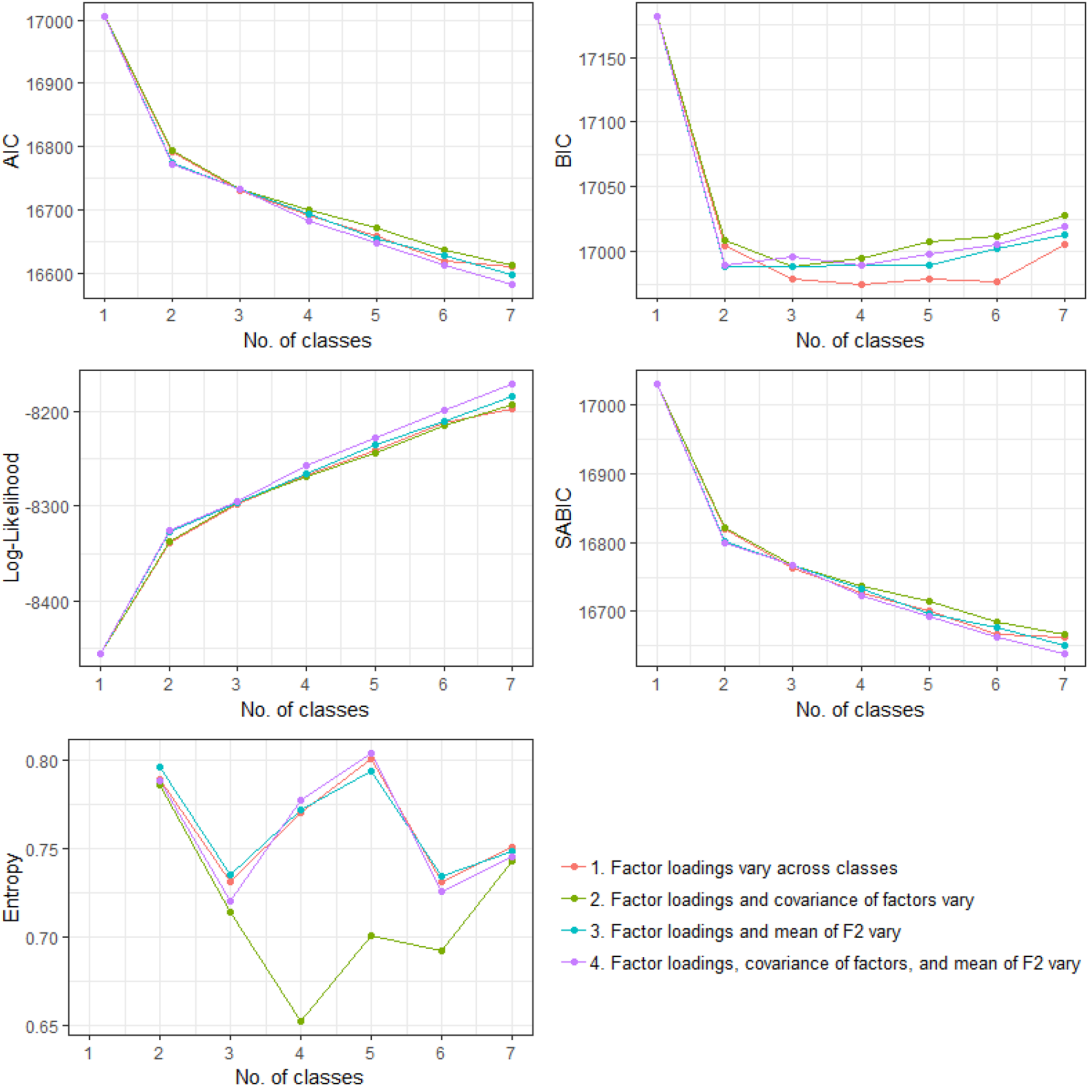
Information criteria (AIC, BIC, SABIC), log-likelihood, and entropy fit indices from the various models. Low values of AIC, BIC, and SABIC should be interpreted as signs of superior model fit, while higher values of entropy and log-likelihood suggest superior fit. Note that a model with only factor loadings varying across classes (1, red line) was selected. Here, entropy appears to favour a two-class solution and BIC a three-class solution, while other fit indices appear to suggest continued improvements through to seven classes

As can be seen in Fig. 2, fit indices generally suggested that there was no substantial improvement to fit from allowing covariances of factors or the mean of F2 to vary across classes, over and above allowing factor loadings to vary across classes. For this reason, all models except those where only the factor loadings were allowed to vary across classes were discarded.

However, fit indices offered somewhat unclear feedback regarding the “optimal” number of classes when only factor loadings were allowed to vary across classes (Fig. 2). Smaller values of AIC, BIC, and SABIC are thought to indicate better model fit, and both AIC and SABIC continuously improved as the number of classes was increased from one to seven. On the other hand, entropy became poorer as the number of classes was increased from two to three (higher entropy values suggest better fit). The BLRT test indicated improvements in fit as the number of classes was increased from one to two, *p* < 0.0001, and from two to three, *p* < 0.0001, but the LMR and VLMR tests found no improvement in fit even from one to two classes, with each yielding *p* = 0.26. Meanwhile, BIC improved substantially when the number of classes was increased from one to two, and there was a further modest improvement of BIC from two to three classes, but BIC afterwards plateaued. Overall, the three-class solution may represent somewhat of an intermediate position between indices favouring a small number of classes (LMR, VLMR) and those favoring a large number (AIC, SABIC). Furthermore, the three-class solution appeared to be clinically and practically meaningful. Therefore, the three-class solution was selected. However, as discussed previously, multiple solutions with varying numbers of classes might each independently convey meaningful information about dimensional variability in these data. We have therefore presented two- and four-class solutions for reference in supplementary materials.

### Description of Classes

In the three-class solution, the first class was characterized by an extremely high loading of the low energy/weak subscale (LEW) onto the latent factor (Table 3), so that, in this class, participants’ overall level of reported sensory behaviour as reflected in the latent factor was highly influenced by raw scores on the LEW subscale (Supplementary Fig. 1). Given the importance of the LEW subscale in shaping the latent overall factor in class 1-LEW, and in order to better understand this subscale, associations between the LEW subscale and other variables were explored in supplementary materials (Supplementary Tables 5 and 6).

**Table 3:** Estimated factor loadings, along with associated standard errors, from each class in the final three-class model

	Class 1–LEW Estimate (*SE*)			Class 2–GPL Estimate (*SE*)			Class 3–NL Estimate (*SE*)		
	Raw	Standardized		Raw	Standardized		Raw	Standardized	
		Time 1	Time 3		Time 1	Time 3		Time 1	Time 3
LEW	11.164 (0.819)	0.987 (0.004)	0.966 (0.015)	2.331 (0.873)	0.791 (0.127)	0.614 (0.193)	– 0.373 (0.183)	– 0.202 (0.078)	– 0.123 (0.066)
TSS	1.598 (0.723)	0.339 (0.137)	0.340 (0.137)	4.217 (0.893)	0.689 (0.082)	0.691 (0.087)	– 2.988 (0.299)	– 0.558 (0.047)	– 0.560 (0.050)
HYI	1.941 (0.624)	0.552 (0.129)	0.503 (0.122)	3.901 (0.621)	0.799 (0.049)	0.760 (0.057)	– 4.181 (0.247)	– 0.819 (0.023)	– 0.782 (0.034)
TS	1.580 (0.419)	0.572 (0.106)	0.618 (0.102)	3.964 (0.641)	0.869 (0.040)	0.892 (0.037)	– 1.442 (0.156)	– 0.538 (0.047)	– 0.583 (0.046)
MS	1.771 (0.361)	0.735 (0.095)	0.764 (0.084)	3.399 (0.706)	0.901 (0.052)	0.916 (0.043)	– 0.140 (0.119)	– 0.085 (0.065)	– 0.093 (0.075)
AD	1.770 (0.358)	0.710 (0.074)	0.634 (0.088)	3.215 (0.503)	0.878 (0.039)	0.830 (0.045)	– 1.420 (0.185)	– 0.629 (0.049)	– 0.549 (0.063)
HRS	0.804 (0.235)	0.499 (0.118)	0.492 (0.112)	1.544 (0.363)	0.742 (0.086)	0.735 (0.080)	– 1.840 (0.112)	– 0.796 (0.030)	– 0.791 (0.031)
VS	1.612 (0.355)	0.715 (0.081)	0.662 (0.085)	1.848 (0.440)	0.761 (0.081)	0.711 (0.090)	– 0.653 (0.127)	– 0.383 (0.063)	– 0.337 (0.060)
ND	0.933 (0.229)	0.472 (0.093)	0.424 (0.096)	2.020 (0.453)	0.757 (0.080)	0.712 (0.080)	– 0.881 (0.174)	– 0.451 (0.068)	– 0.404 (0.079)

Factors are: Low Energy/Weakness (LEW), Taste/Smell Sensitivity (TSS), Hyperactivity/Inattention (HYI), Tactile Sensitivity (TS), Movement Sensitivity (MS), Auditory Distractibility (AD), Hyporesponsiveness to Speech (HRS), Visual Sensitivity (VS), and Noise Distress (ND). Note that raw factor loadings were held equal across time points by the model, but standardized loadings show slight differences from Time 1 to Time 3

The second class of participants had more balanced loadings, and it is thus referred to as the Generalized Positive Loadings (GPL) class. These loadings appeared to reflect these participants’ problematic reported sensory features and behaviours across a number of different subscales. These were most notably Taste/Smell Sensitivity (TSS), Hyperactivity/Inattention (HYI), Tactile Sensitivity (TS), Movement Sensitivity (MS) and Auditory Distractibility (AD).

Finally, the third class was characterized by negative loadings of the subscales onto the latent factor, and it is referred to as the Negative Loadings (NL) class. These loadings appeared to reflect the presence of relatively few unusual or problematic sensory features, with notable negative loadings on HYI and TSS.

Average posterior probabilities of individuals’ class assignments are given in Table 4. The lowest average posterior probability was 89%, suggesting class assignments were, for the most part, quite confident.

**Table 4:** Posterior probabilities of assignment to each class, as averaged across all participants in each class

		Actual Assigned Class		
		Class 1-LEW (%)	Class 2-GPL (%)	Class 3-NL (%)
Probability of Assignment to…	Class 1-LEW	92.09	1.22	4.46
	Class 2-GPL	3.45	91.38	6.09
	Class 3-NL	4.46	7.40	89.46

Columns define actual class assignments, while rows refer to the posterior probabilities of assignment within each class for participants in a given column. That is, participants who are actually assigned to Class 1-LEW have on average a 92.09% posterior probability of assignment to said class, along with a 3.45% posterior probability of assignment to Class 2-GPL and a 4.46% posterior probability of assignment to Class 3-NL

### Classes and Diagnostic Groups

The 1-LEW and 2-GPL classes were overwhelmingly dominated by autistic participants, while the 3-NL class was a mixed group of typically-developing and autistic participants (Table 5). A Fisher’s exact test indicated that the proportions of participants from each diagnostic group were not homogeneous across classes, *p* < 0.0001.

**Table 5:** Count of participants in each class and diagnostic group, as well as percentage of participants from each diagnostic group assigned to a given class

	Class 1-LEW		Class 2-GPL		Class 3-NL	
	ASD	TD	ASD	TD	ASD	TD
Count	53	2	24	1	113	92
Percentage	27.89%	2.11%	12.63%	1.05%	59.47%	96.84%

**Table 6:** Count and proportion of participants from each class and diagnostic group with usable SSP data at each time point

	Class 1-LEW		Class 2-GPL		Class 3-NL	
	ASD	TD	ASD	TD	ASD	TD
Time 1	48 (90.57%)	2 (100.00%)	20 (83.33%)	1 (100.00%)	104 (92.04%)	84 (91.30%)
Time 3	31 (58.49%)	0 (0.00%)	14 (58.33%)	1 (100.00%)	42 (37.17%)	54 (58.70%)

Interestingly, a Fisher’s test comparing participants from the four largest combinations of class and diagnostic group (i.e., excluding TD participants in classes 2 and 3) found a lack of homogeneity in the proportions of participants with and without Time 3 SSP data, *p* = 0.006.

### Classes and Raw SSP Scores

As very few typically-developing participants were assigned to classes 1-LEW and 2-GPL (Table 6), analyses comparing classes on the basis of raw SSP scores were carried out within the ASD group. Participants assigned to separate classes differed in the raw scores they obtained on a number of SSP subscales at both Time 1 (Table 7) and Time 3 (Table 8), confirming that differences in factor loadings translated into differences in actual scores. Visualizations of participant raw scores and trajectories on each subscale are available in supplementary materials (Supplementary Figs. 1, 2, 3, 4, 5, 6, 7, 8, and 9), as are longitudinal comparisons of SSP subscores at Times 1 and 3 within each class and group (Supplementary Tables 7, 8, 9, and 10).

**Table 7:** At Time 1, mean and standard deviations of SSP subscale scores from autistic participants assigned to each class, along with results of Kruskal–Wallis omnibus tests and corrected post-hoc tests

	Mean (SD) By Class			Kruskal–Wallis Omnibus Test			Cliff’s δ Post-Hoc Effect Size^a^ with 95% CI		
	1-LEW	2-GPL	3-NL	χ^2^	*p*	*η*^2^	LEW vs. GPL	LEW vs. NL	GPL vs. NL
LEW	18.90 (6.45)	27.05 (3.32)	28.83 (1.84)	84.54	< .0001	.49	− .74*** [− .87, − .52]	− .87*** [− .94, − .73]	− .31* [− .55, − .01]
TSS	12.52 (5.07)	9.85 (5.71)	12.65 (5.57)	4.45	.11	.02	.28 [− .05, .56]	− .02 [− .21, .17]	− .29 [− .54, − .00]
HYI	13.67 (3.65)	11.90 (3.19)	14.45 (4.59)	7.36	.03	.03	.29 [− .02, .55]	− .12 [− .30, .07]	− .36* [− .56, − .12]
TS	15.57 (3.22)	12.30 (3.42)	16.90 (2.65)	27.84	< .0001	.16	.53** [.26, .72]	− .24* [− .42, − .04]	− .71*** [− .83, − .52]
MS	12.38 (1.85)	9.85 (3.15)	14.23 (1.26)	61.53	< .0001	.35	.49** [.15, .72]	− .59*** [− .73, − .41]	− .80*** [− .92, − .53]
AD	11.32 (2.12)	9.30 (3.44)	12.05 (2.40)	14.99	.0006	.08	.37* [.01, .64]	− .23* [− .41, − .04]	− .48** [− .70, − .16]
HRS	4.92 (1.37)	3.75 (1.62)	4.90 (1.67)	9.79	.007	.05	.44** [.13, .67]	.01 [− .18, .19]	− .40** [− .62, − .13]
VS	6.89 (2.42)	6.15 (2.43)	8.31 (1.70)	21.00	< .0001	.11	.18 [− .13, .45]	− .34** [− .52, − .14]	− .52*** [− 72, − .23]
ND	7.46 (1.54)	5.15 (2.43)	7.95 (2.14)	23.36	< .0001	.13	.57*** [.22, .79]	− .24* [− .41, − .06]	− .59*** [− .78, − .31]

Values of δ are denoted with * if the corresponding Wilcoxon-Mann–Whitney *p* value is < .05 after Bonferroni-Holm correction for three comparisons, with ** if *p* < .01, and with *** if *p* < .001

**Table 8:** At Time 3, mean and standard deviations of SSP subscale scores from autistic participants assigned to each class, along with results of Kruskal–Wallis omnibus tests and corrected post-hoc tests

	Mean (SD) By Class			Kruskal–Wallis Omnibus Test			Cliff’s δ Post-Hoc Effect Size^a^ with 95% CI		
	1-LEW	2-GPL	3-NL	χ^2^	*p*	*η*^2^	LEW vs. GPL	LEW vs. NL	GPL vs. NL
LEW	16.61 (5.99)	23.79 (6.03)	28.14 (2.83)	48.65	< .0001	.56	− .58** [− .80, − .22]	− .93*** [− .97, − .82]	− .42* [− .70, − .02]
TSS	12.47 (4.88)	10.79 (6.02)	12.56 (5.42)	0.98	.61	.01	.19 [− .22, .55]	− .04 [− .30, .23]	− .14 [− .49, .24]
HYI	14.81 (4.13)	9.71 (2.64)	14.76 (4.79)	15.06	.0005	.16	.71*** [.43, .86]	− .03 [− .29, .24]	− .62** [− .79, − .35]
TS	16.45 (2.38)	13.79 (3.66)	17.63 (2.22)	14.44	.0007	.15	.44* [.01, .74]	− .32* [− .55, − .05]	− .60** [− .83, − .20]
MS	12.19 (2.51)	10.23 (2.24)	14.61 (0.77)	40.83	< .0001	.47	.46* [.11, .70]	− .62*** [− .78, − .38]	− .96*** [− .99, − .84]
AD	9.32 (2.63)	8.64 (1.69)	11.17 (2.65)	15.04	.0005	.16	.14 [− .20, .45]	− .41** [− .62, − .13]	− .60** [− .78, − .33]
HRS	5.45 (1.67)	5.00 (1.66)	5.95 (1.77)	4.29	.12	.03	.13 [− .24, .47]	− .20 [− .44, .07]	− .32 [− .60, .02]
VS	7.52 (2.20)	7.07 (2.34)	8.39 (1.96)	5.97	.05 (*n*.*s*.)	.05	.13 [− .27, .49]	− .27 [− .50, − .01]	− .33 [− .63, .05]
ND	6.06 (2.14)	6.07 (2.34)	6.60 (2.29)	1.01	.60	.01	− .03 [− .39, .34]	− .13 [− .38, .14]	− .12 [− .44, .23]

^a^Values of δ are denoted with * if the corresponding Wilcoxon–Mann–Whitney *p* value is < .05 after Bonferroni-Holm correction for three comparisons, with ** if *p* < .01, and with *** if *p* < .001; *n.s.* means not significant

In addition, Wilcoxon-Mann–Whitney tests were used to determine whether autistic and typically-developing participants assigned to class 3-NL significantly differed in their raw SSP scores at Time 1 and also at Time 3. As shown in Table 9, autistic participants in this class had lower (more atypical) scores than their typically-developing counterparts on almost all subscales.

**Table 9:** SSP raw scores of ASD and TD participants assigned to class 3-NL at each time point, along with p-values from Wilcoxon-Mann–Whitney tests and Cliff’s δ effect size measurements comparing scores from each diagnostic group

	Time 1					Time 3				
	Mean (SD)		*p*	Cliff’s δ		Mean (SD)		*p*	Cliff’s δ	
	ASD	TD		Value	95% CI	ASD	TD		Value	95% CI
LEW	28.83 (1.84)	29.75 (0.92)	< .0001	− .29	− .40, − .17	28.14 (2.83)	29.43 (1.09)	.05 (*n*.*s*.)^a^	− .19	− .38, .01
TSS	12.65 (5.57)	17.95 (2.70)	< .0001	− .54	− .66, − .39	12.56 (5.42)	18.15 (3.24)	< .0001	− .67	− .80, − .47
HYI	14.46 (4.59)	19.70 (3.19)	< .0001	− .64	− .75, − .51	14.76 (4.79)	21.59 (3.23)	< .0001	− .76	− .86, − .59
TS	16.90 (2.65)	18.81 (1.65)	< .0001	− .46	− .59, − .31	17.63 (2.22)	19.31 (1.36)	< .0001	− .57	− .73, − .36
MS	14.23 (1.26)	14.13 (1.45)	.67	.03	− .12, .18	14.61 (0.77)	14.44 (1.11)	.56	.06	− .13, .24
AD	12.05 (2.40)	13.93 (1.17)	< .0001	− .51	− .63, − .36	11.17 (2.65)	13.69 (1.70)	< .0001	− .61	− .76, − .40
HRS	4.90 (1.67)	8.24 (1.49)	< .0001	− .84	− .90, − .74	5.95 (1.77)	8.28 (1.45)	< .0001	− .69	− .81, − .51
VS	8.31 (1.70)	8.92 (1.17)	.03	− .18	− .33, − .02	8.39 (1.96)	9.04 (1.50)	.09	− .19	− .40, .04
ND	7.95 (2.14)	8.21 (1.65)	.75	− .03	− .19, .14	6.60 (2.29)	8.52 (1.83)	< .0001	− .51	− .68, − .28

^a^*n.s.* means not significant

### Classes and Chronological Age in ASD

**Fig. 3:**
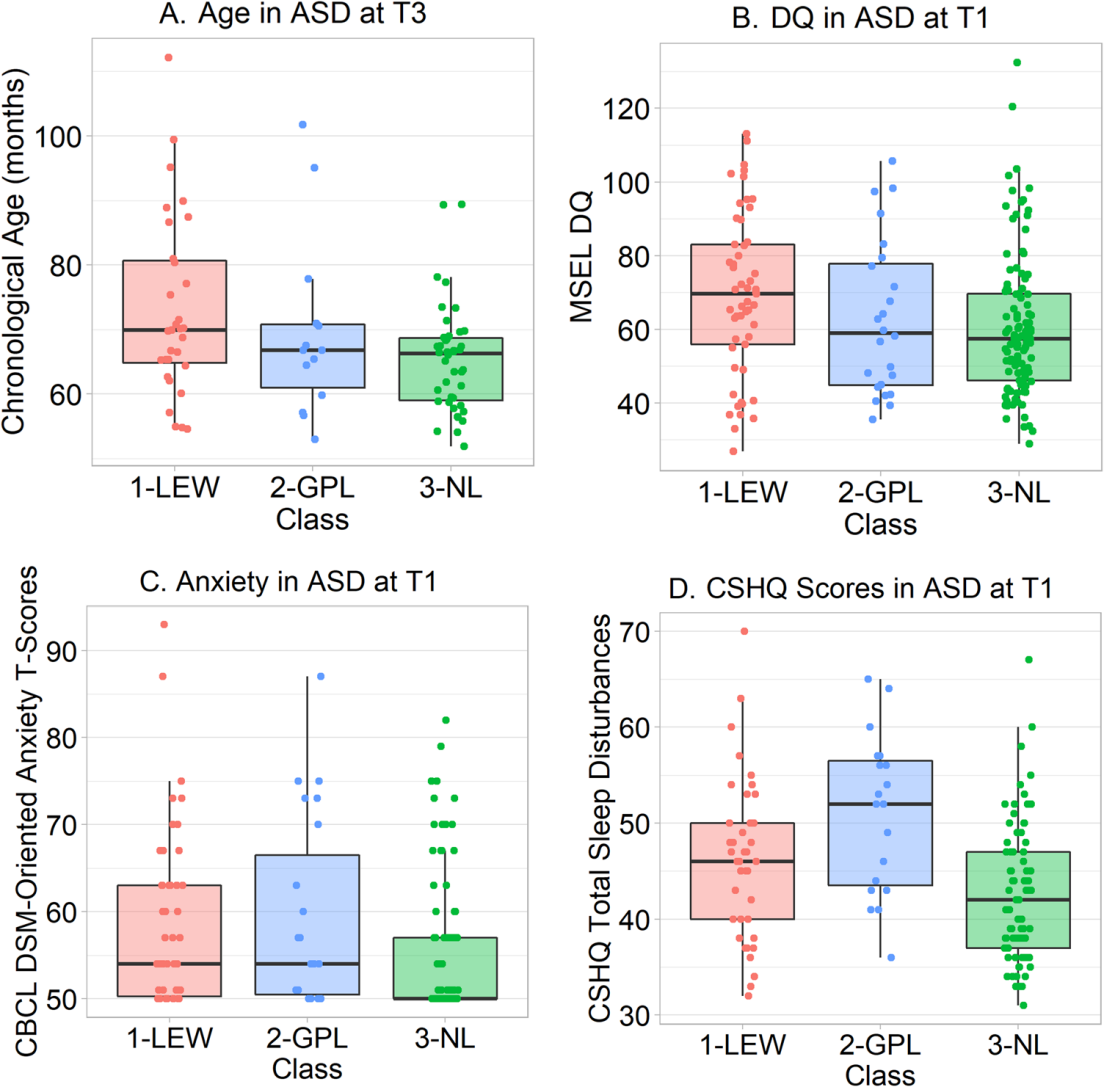
Boxplots comparing autistic participants assigned to different classes, overlaid by individual participants’ scores. From left to right and top to bottom: **A** Chronological age of autistic participants in months at Time 3; **B** MSEL DQ of autistic participants at Time 1; **C** CBCL DSM-oriented anxiety T-scores of autistic participants at Time 1; **D** CSHQ total sleep disturbances scores of autistic participants at Time 1

Chronological age did not differ across autistic participants assigned to separate classes at Time 1, Kruskal–Wallis χ^2^ = 0.22, *p* = 0.89, *η*^2^ = 0.01 (Supplementary Table 11). At Time 3, a trend towards an effect did not attain statistical significance, Kruskal–Wallis χ^2^ = 5.78, *p* = 0.06, *η*^2^ = 0.05 (Fig. 3A; Supplementary Table 12). This trend was driven by older age in class 1-LEW than class 3-NL, an effect which at post hoc remained significant after Bonferroni-Holm correction for three comparisons, Wilcoxon *p* = 0.04, δ = 0.34 [95% CI 0.06, 0.57].

### Classes and Cognitive Ability in ASD

At Time 1, MSEL DQ significantly differed across autistic participants assigned to separate classes, Kruskal–Wallis χ^2^ = 7.70, *p* = 0.02, *η*^2^ = 0.03 (Fig. 3B; Supplementary Table 11). This was driven by higher MSEL DQ scores in class 1-LEW (*M* = 69.26, *SD* = 0.21.83) than class 3-NL (*M* = 60.42, *SD* = 19.12), corrected Wilcoxon *p* = 0.01, δ = 0.27 [95% CI 0.07, 0.46]. DAS GCA scores did not differ across classes at Time 3, Kruskal–Wallis χ^2^ = 1.33, *p* = 0.51, *η*^2^ = 0.01 (Supplementary Table 12).

### Classes and Adaptive Behaviour in ASD

VABS composite scores did not significantly differ across autistic participants assigned to separate classes at Time 1, Kruskal–Wallis χ^2^ = 0.33, *p* = 0.85, *η*^2^ = 0.01 (Supplementary Table 11), or Time 3, Kruskal–Wallis χ^2^ = 0.37, *p* = 0.83, *η*^2^ = 0.02 (Supplementary Table 12).

### Classes and Anxiety in ASD

At Time 1, CBCL DSM-oriented anxiety T-scores significantly differed across autistic participants assigned to separate classes, Kruskal–Wallis χ^2^ = 15.09, *p* = 0.0005, *η*^2^ = 0.07 (Fig. 3C; Supplementary Table 11). This omnibus effect was driven by higher anxiety in class 1-LEW than class 3-NL, corrected Wilcoxon *p* = 0.002, δ = 0.31 [95% CI 0.13, 0.48], and in class 2-GPL than class 3-NL, corrected Wilcoxon *p* = 0.01, δ = 0.34 [95% CI 0.09, 0.55]. At Time 3, a trend towards an effect did not attain statistical significance, Kruskal–Wallis χ^2^ = 5.33, *p* = 0.07, *η*^2^ = 0.04 (Supplementary Table 12). The strongest post hoc trend at Time 3–viz., one towards higher anxiety in class 2-GPL than class 3-NL–did not approach significance after Bonferroni-Holm correction, corrected Wilcoxon *p* = 0.15, δ = 0.33 [95% CI –0.02, 0.61].

### Classes and Sleep in ASD

At Time 1, CSHQ total sleep disturbance scores significantly differed across autistic participants assigned to separate classes, Kruskal–Wallis χ^2^ = 17.82, *p* = 0.0001, *η*^2^ = 0.11 (Fig. 3D; Supplementary Table 11). Caregivers of participants assigned to class 2-GPL, corrected Wilcoxon *p* = 0.0003, δ = 0.57 [95% CI 0.30, 0.76], and to class 1-LEW, corrected Wilcoxon *p* = 0.03, δ = 0.28 [95% CI 0.04, 0.48], reported more sleep disturbances than caregivers of those assigned to class 3-NL. Furthermore, and interestingly, caregivers of participants in class 2-GPL reported significantly more sleep problems than those in class 1-LEW, corrected Wilcoxon *p* = 0.05, δ = 0.33 [95% CI –0.00, 0.59]. At Time 3, no effect of CSHQ total scores was evident, Kruskal–Wallis χ^2^ = 2.03, *p* = 0.36, *η*^2^ = 0.04 (Supplementary Table 12).

### Classes and ERPs in ASD

#### Latency

**Fig. 4:**
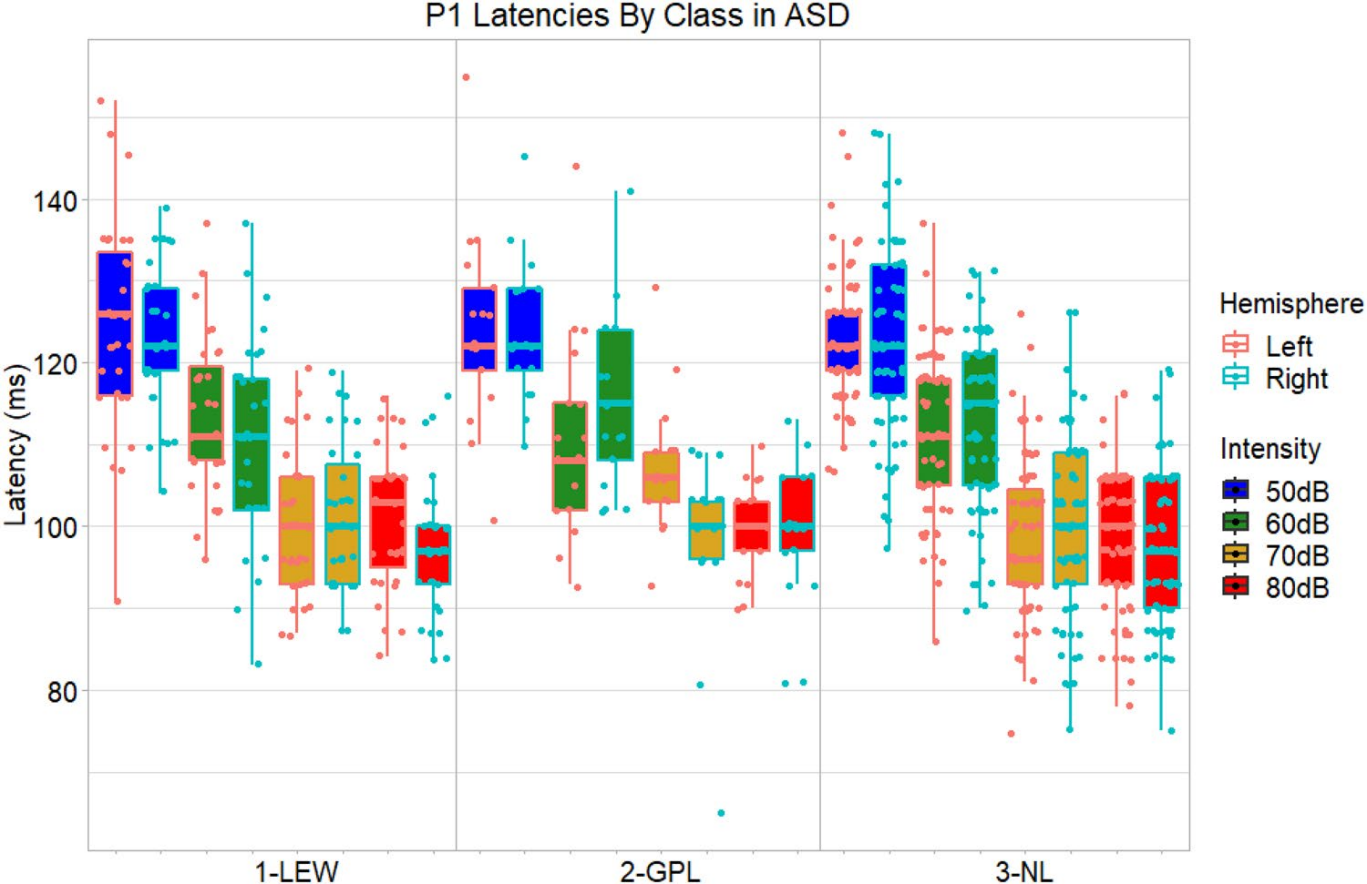
Boxplots comparing P1 ERP latencies across autistic participants assigned to different classes, overlaid by individual participants’ latency values. A significant three-way ANOVA interaction of stimulus intensity (loudness), hemisphere, and mixture model class was observed. Uncorrected tests suggested P1 latencies might be shorter over the right hemisphere in class 1-LEW in the 80 dB condition as well as in class 2-GPL in the 70 dB condition, but these effects were modest and did not survive correction for multiple comparisons

Although a main effect of stimulus intensity (loudness) on P1 latency was observed, *F*(3, 336) = 292.22, *p* < 0.0001, ${\eta }_{G}^{2}$ = 0.43 (Fig. 4; Supplementary Table 13), no main effects or two-way interactions involving class approached significance in ASD. There was a significant three-way interaction of class, intensity, and hemisphere on latency, *F*(6, 336) = 2.78, *p* = 0.01, ${\eta }_{G}^{2}$ = 0.01. However, no post-hoc effects were strong enough to survive correction for multiple comparisons. Thus, it is not clear that meaningful P1 latency differences emerged between classes.

#### Amplitude

**Fig. 5:**
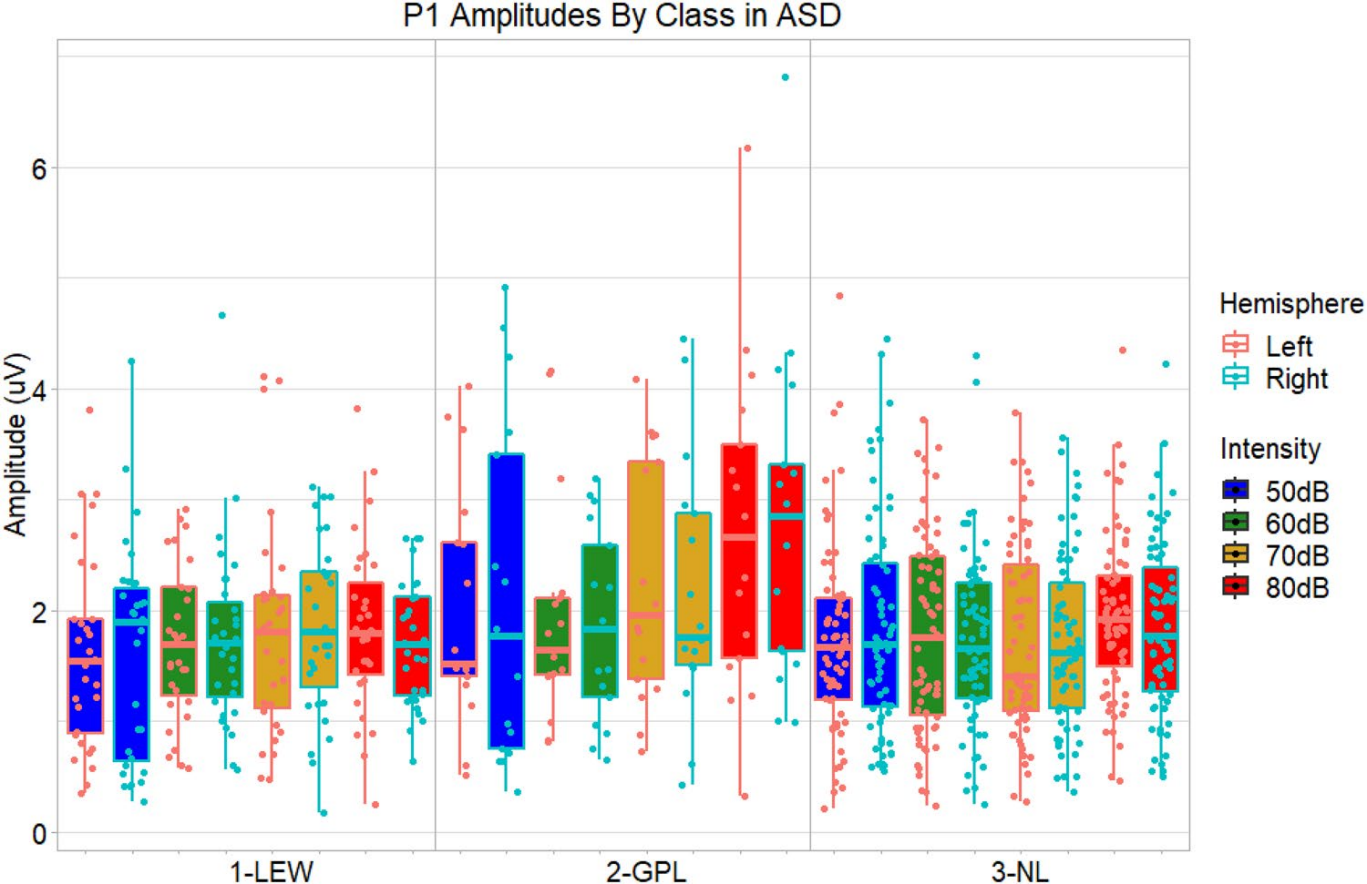
Boxplots comparing P1 ERP amplitudes across autistic participants assigned to different classes, overlaid by individual participants’ amplitude values. Note that P1 amplitudes in the 80 dB condition remained significantly larger in 2-GPL than other classes after correction for multiple comparisons and after removal of the outlying participant in class 2-GPL (viz., the participant with amplitude > 6 μV)

**Fig. 6:**
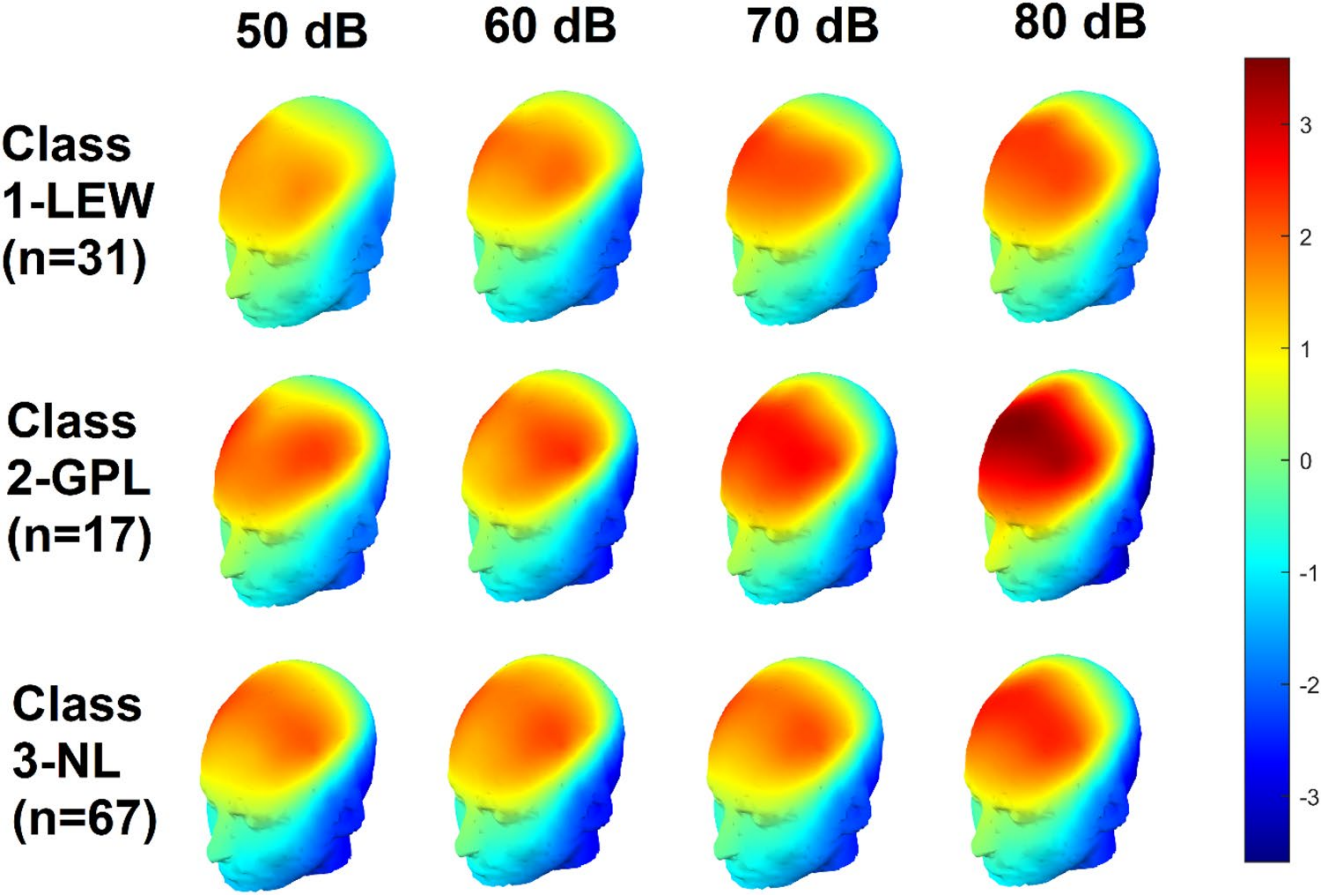
Scalp plots depicting spherically-splined P1 auditory response voltages of autistic participants in each class during separate 51 ms time windows from the centre of the P1 measurement window in each condition, or 95–145 ms (50 dB), 84–134 ms (60 dB), 69–119 ms (70 dB), and 66–116 ms (80 dB). The conditions are arrayed in columns from left to right (i.e., 50 dB at left; 80 dB at right), while the classes are arrayed in rows: 1-LEW at top, 2-GPL in middle, and 3-NL at bottom. The scale in microvolts (μV) is given at the far right. Note the increased response amplitude at 80 dB for the 2-GPL class

Significant main effects of class, *F*(2, 112) = 4.24, *p* = 0.02, ${\eta }_{G}^{2}$ = 0.03, and stimulus intensity, *F*(3, 336) = 5.69, *p* = 0.0008, ${\eta }_{G}^{2}$ = 0.02, on P1 amplitude were observed (Figs. 5, 6, Table 10). The main effect of intensity remained significant after Greenhouse–Geisser correction, GGε = 0.88, *p* = 0.001. Amplitudes were higher to 80 dB sounds than all other sounds, Bonferroni-Holm corrected *p* ≤ 0.02. Furthermore, follow-up ANOVAs comparing pairs of classes indicated that ERP amplitudes were generally higher in class 2-GPL than class 1-LEW, *F*(1, 82) = 6.39, Bonferroni-Holm corrected *p* = 0.03, ${\eta }_{G}^{2}$ = 0.06, and in class 2-GPL than class 3-NL, *F*(1, 82) = 6.27, corrected *p* = 0.04, ${\eta }_{G}^{2}$ = 0.04.

**Table 10:** At Time 1, in autistic participants only, means and standard deviations of P1 area amplitude over each hemisphere and for each intensity level and SSP-derived class

	Class 1-LEW Mean (SD)		Class 2-GPL Mean (SD)		Class 3-NL Mean (SD)	
	Left	Right	Left	Right	Left	Right
50 dB	1.62 (0.87)	1.62 (0.99)	2.02 (1.07)	2.08 (1.52)	1.62 (0.99)	1.85 (0.97)
60 dB	1.71 (0.69)	1.75 (0.81)	1.95 (1.00)	1.86 (0.85)	1.78 (0.89)	1.72 (0.79)
70 dB	1.81 (0.97)	1.84 (0.77)	2.26 (1.08)	2.16 (1.14)	1.73 (0.85)	1.72 (0.78)
80 dB	1.84 (0.76)	1.70 (0.56)	2.70 (1.44)	2.81 (1.50)	1.95 (0.73)	1.85 (0.79)

In addition, however, a two-way interaction between class and intensity attained significance, *F*(6, 336) = 2.38, *p* = 0.03, ${\eta }_{G}^{2}$ = 0.01 (Supplementary Fig. 10), and this interaction remained significant after Greenhouse–Geisser correction for sphericity violation, GGε = 0.88, *p* = 0.04. Due to the presence of this interaction, the original omnibus main effects of class and intensity should be regarded with caution. Follow-up ANOVAs comparing pairs of classes at each intensity level indicated that ERP amplitudes in autistic participants, after Bonferroni-Holm correction for twelve comparisons, were only significantly higher in class 2-GPL than class 1-LEW, and than class 3-NL, in the 80 dB condition (Table 11).

**Table 11:** At Time 1, in autistic participants only, main effects of class in 2 × 2 follow-up mixed ANOVAs with P1 area amplitude as the dependent variable, comparing each pair of classes as the between-subjects variable, and with hemisphere as the within-subjects variable

	1-LEW vs. 2-GPL				1-LEW vs. 3-NL				2-GPL vs. 3-NL			
	*F*(1,46)	*p*	*p*(cor)	${\eta }_{G}^{2}$	*F*(1,96)	*p*	*p*(cor)	${\eta }_{G}^{2}$	*F*(1,82)	*p*	*p*(cor)	${\eta }_{G}^{2}$
50 dB	2.08	.16	N/A	.04	0.94	.34	N/A	.01	1.14	.29	N/A	.01
60 dB	0.60	.44	N/A	.01	0.02	.89	N/A	.00	0.52	.47	N/A	.00
70 dB	2.13	.15	N/A	.04	0.38	.54	N/A	.00	5.03	.03	.28	.05
80 dB	11.88	.001	.01	.18	0.95	.33	N/A	.01	13.56	.0004	.005	.12

To aid in statistical inference, p-values are provided both without correction and, for values that attained significance before correction, with Bonferroni-Holm corrections for twelve multiple comparisons

## Discussion

The present study found clear evidence of meaningful heterogeneity of SSP scores within autism. A total of three subgroups were described, each with different loadings of SSP subscales onto latent factors. The significance and meaningfulness of these subgroups is emphasized by findings that cognitive ability, anxiety, sleep quality, and auditory event-related potential amplitudes differed across autistic participants assigned to separate classes.

Notably, allowing levels of the latent factor to vary over time did not appear to improve model fit, in contrast to results obtained by Dwyer et al. (2020a) in the same sample. This may reflect the very limited number of participants with atypical trajectories observed by Dwyer et al., coupled with the greater complexity of the factor mixture model used in the present study.

### Class 3-NL

As predicted by the first hypothesis, almost all participants in the TD group were assigned to a single class: namely, class 3-NL. The first hypothesis accurately predicted that many autistic participants would be assigned to this class as well, although the fact that the *majority* of autistic participants would be assigned to the class was not necessarily anticipated. However, it would not be appropriate to say that autistic participants in class 3-NL had typical sensory processing, or that class 3-NL is defined by typical sensory processing per se. Indeed, autistic participants in class 3-NL were found to have significantly lower (i.e., more atypical/problematic) scores than typically-developing participants on every SSP subscale except for the one indexing movement sensitivity at one or both of the time points from the present study. The autistic participants in class 3-NL did have higher SSP scores on many subscales than autistic participants in other classes, but their SSP scores clearly differed from those of most TD participants. It might be more accurate to say that SSP subscales of autistic participants assigned to class 3-NL covaried with the latent factors in a manner more similar to the bulk of typically-developing participants than to autistic participants in other classes. The overall level of sensory processing indexed by the latent factor itself could still be atypical in some participants from class 3-NL.

### Class 1-LEW

The second hypothesis predicted that many autistic participants would be assigned to other classes. This too was supported. Two other classes besides class 3-NL were described: class 1-LEW and class 2-GPL. Factor loadings indicate that the LEW subscale of the SSP exercised an outsized influence over the overall SSP performance of participants in class 1-LEW, and indeed, autistic participants in class 1-LEW showed markedly lower raw SSP LEW scores than autistic participants in other classes. Its dominant role in this subgroup emphasizes the importance of understanding the LEW subscale, as does the fact that the LEW subscale is found not only in the factors defined by Williams et al., (2018a, b), but also the subscales presented by McIntosh et al. (1999) and Tomchek et al. (2014). Prior studies also suggest the LEW subscale may have a large role in sensory heterogeneity in ASD (Hand et al., 2017; Lane et al., 2014).

Exploratory analyses presented in supplementary materials do note some associations between LEW scores and other SSP subscales, which might imply that the LEW subscale is partly related to individual differences in overall sensory processing. However, correlations between the LEW subscale and the SSP were generally fairly modest, such that it may not fully or even primarily index sensory processing. Furthermore, the LEW scale does not seem to primarily measure sleep; notably, autistic participants in the LEW class had better sleep quality than those in class 2-GPL.

Another possibility may be that LEW scale taps into hypotonia and/or physical inactivity. Analyses suggested that lower levels of energy and higher levels of weakness on the SSP were associated with worse fine and gross motor adaptive skills (as measured on the VABS). Although prior research did not find significant correlations between LEW scores and motor performance in ASD (Tomchek et al., 2015), prior research suggests physical activity levels and motor skills may be independent in autistic preschoolers (Ketcheson et al., 2018), which raises intriguing questions regarding what factors might drive individual differences in physical activity in these children. Further research may be necessary to explore that question, as well as whether the LEW scale might be more associated with autistic children’s actual levels of exercise and mobility than with their motor skills.

Interestingly, low (more atypical/problematic) LEW scores were also related to increased verbal and nonverbal cognitive abilities at Time 1, and indeed, autistic participants in class 1-LEW were observed to have higher MSEL DQ at Time 1 than participants in other classes. Tomchek et al. (2015) might be read as implying that sedentary behaviour in ASD could be linked to better opportunities for language learning.^3^ If this is the case, it is notable that LEW scores in the present sample were far lower (i.e., more “atypical”) in class 1-LEW autistic participants than typically-developing participants; the LEW phenotype could therefore represent a protective developmental mechanism in ASD that is not evident in TD. However, the possible benefits of low energy/weakness should not be over-emphasized, as low energy and high weakness were also related to internalizing problems. It is also noteworthy that cognitive ability effects in the present study were not significant at Time 3, though it is possible that this could partly reflect the more limited range of the DAS compared to the MSEL. Moreover, in prior research, autistic toddlers with hypotonia have been reported by parents to have lower quality of life, not only in the physical domain but also psychosocially (Lopez-Espejo et al., 2021). Autistic children with hypotonia also have more autistic characteristics than those without (Lopez-Espejo et al., 2021), and although not all autistic behaviours are weaknesses (Russell et al., 2019), current measures of autistic behaviour are based on a pathology paradigm and accordingly do focus on areas of challenge (Timini et al., 2019). Thus, evidence regarding whether hypotonia and low energy/weakness are protective or problematic seems mixed.

Although the LEW subscale is emphasized in discussion of class 1-LEW due to the sheer extent of its influence, it should be borne in mind that loadings on other subscales were positive, in contrast to the negative loadings in class 3-NL. In keeping with this, autistic participants in class 1-LEW had significantly lower raw scores than those in class 3-NL on tactile sensitivity, movement sensitivity, and auditory distractibility at both time points, as well as visual sensitivity and noise distress at Time 1 only. Thus, class 1-LEW is not solely defined by the LEW subscale.

### Class 2-GPL

However, it appears that class 2-GPL might be relatively more influenced by these other subscales. Class 2-GPL is defined primarily be generalized positive loadings across SSP subscales, with comparatively balanced loadings suggesting no single subscale is largely responsible for this pattern. The LEW subscale aside, autistic participants in class 2-GPL showed the lowest raw SSP scores. Scores were even lower than SSP scores in class 1-LEW on (at both time points) the tactile and movement sensitivity subscales; (at Time 1) the auditory distractibility, hypo-responsiveness to speech, and noise distress subscales; and (at Time 3) the hyperactivity/inattention subscale.

This phenotype of hyper-reactivity, hyper-activity, and at least social/linguistic hypo-responsiveness was linked to P1 auditory ERPs, providing evidence of multimodal convergence of caregiver-report questionnaire patterns with a neurophysiological response. Specifically, among ASD participants, main effects and interactions could be taken to suggest generally higher P1 amplitudes and particularly high amplitudes in the 80 dB condition in class 2-GPL. Admittedly, the main effect should be interpreted with caution due to the interaction, and only one of nine main effects comparing autistic participants in different classes achieved significance outside the 80 dB condition even before multiple comparison correction. However, the interaction effect involving the 80 dB condition appears quite consistent with prior associations observed in the present sample between relative 80 dB response strength in the P1 latency range and auditory distractibility and noise distress (Dwyer et al., 2020b). A novel contribution of the present study is the finding that these patterns appear linked to atypical sensory processing in other modalities besides hearing alone, perhaps due to central nervous system influences on sensory processing (such as attention or excitation-inhibition balances) that might hold across modalities.

### Anxiety and Sleep

Autistic participants in classes 1-LEW and 2-GPL–that is, those with relatively low SSP scores, suggesting more unusual or problematic sensory processing–were found to have significantly more anxiety and more sleep disturbances than autistic children in class 3-NL at Time 1. Differences between classes were not significant at Time 3. As there was still a strong trend towards a between-class difference in anxiety at Time 3, it is not clear that developmental changes removed between-class differences; attrition between Time 1 and Time 3 may simply have reduced power to detect such effects. In any case, the findings at Time 1 appear broadly consistent with prior research linking sensory processing to sleep (Tzischinsky et al., 2018) and anxiety (Mazurek et al., 2013; Neil et al., 2016; Uljarević et al., 2016) in ASD. Prior longitudinal research (Green et al., 2012) suggests that sensory processing differences might have contributed towards the development of anxiety in these individuals. In particular, sensory sensitivities could cause individuals to fear environments or stimuli that might evoke sensory distress. However, it does not seem impossible that the association could also be bidirectional; anxious vigilance might make distressing sensory stimuli more salient. Qualitative research does note that anxiety exacerbates sensory challenges in autism (Landon et al., 2016). Meanwhile, it seems plausible that sensory processing differences could drive later sleep disturbances, but further analyses would be necessary to empirically explore this. Overall, despite the lack of differences in adaptive functioning between autistic participants in different classes, the effects of anxiety and sleep (both of which are relevant to quality of life in ASD; see Adams et al., 2019; Deserno et al., 2019; Smith et al., 2019) emphasize the importance of sensory processing in the lives of autistic people.

### Limitations

The present study has a number of strengths. These notably include its use of a large, well-characterized longitudinal sample, as well as its inclusion of a neurophysiological measure–namely, P1 auditory event-related potentials–to complement caregiver reports of sensory behaviours. However, it is not without limitations.

One limitation is the loss of some participants between Time 1 and Time 3 of the study. Although some level of attrition is only to be expected in a longitudinal study, it is noteworthy that autistic participants in class 3-NL seemed to be less likely to be retained at Time 3. It is not impossible that the factor loadings characteristic of class 3-NL were relatively uncommon in ASD at Time 3, such that the fit of the classes may have differed across time points and groups.

Although the present study sample is large, the computational demands of the factor mixture modelling approach used here prevented us from splitting the sample or using resampling to evaluate the stability of the classes. This makes it difficult to claim that the classes described in the present study exist as clear categorical groups that could be reliably replicated by future studies using similar methods; however, we view these classes primarily as a descriptive technique for exploring data patterns and variability. We remain open to the possibility that other subgrouping solutions might illuminate different patterns and variability.

Another limitation of the present study is the lack of multimodal measures indexing non-auditory domains of sensory processing, such as touch or vision. Although auditory neurophysiological hyper-reactivity to loud sounds converged in the present study with a caregiver-reported phenotype of sensory sensitivity, as well as hyporesponsiveness to speech and hyperactivity/inattention, the present study cannot establish whether and how caregiver reports of sensory behaviours from the present study would have converged with neurophysiological measures outside the auditory modality. In addition, the present study relies on only a single caregiver-report questionnaire, the SSP, and thus lacks the “parallel validation” that could be offered by including other similar measures (Agelink van Rentergem et al., 2021). We also lack other relevant types of sensory measure, such as perceptual acuity measures.

Furthermore, although the present study indicated that autistic participants’ levels of anxiety and sleep differed across classes, suggesting associations between these variables and sensory processing, the present study does not resolve the directionality these associations. Additionally, the present study does not determine whether specific SSP subscales accounted for a relatively larger degree of variance in anxiety and sleep than other subscales. Thus, further research will be necessary to explore associations between sensory processing scores and the variables of anxiety and sleep.

One final limitation of the present study is that the SSP subscale solution proposed by Williams et al., (2018a, b) and employed here has not been tested for measurement invariance across samples of autistic and non-autistic children. However, the factor mixture modelling approach used in this study arguably is in some ways related to questions of measurement invariance, insofar as it examines inter-individual differences in how the separate SSP factors converge with overall SSP performance. Indeed, whereas measurement invariance analyses may in practice be used to justify the exclusion or elimination of particular subscales that might, due to their very differential function across groups, be of considerable substantive interest, the present study’s approach simply highlights subgroups of individuals whose SSP scores on a particular subscale contribute to overall SSP performance in a manner disproportionate to other subgroups.

## Summary

The present study suggests that there are multiple different patterns of relative contributions of different SSP subscales towards overall SSP performance in ASD, as indicated by differences in factor loadings across classes. The present study also finds a single pattern–that of class 3-NL–that is characteristic of most typically-developing individuals. The largest single group of autistic participants also exhibited this pattern, as reflected by their membership in class 3-NL, although autistic participants in this class still had lower mean SSP scores on most subscales than their typically-developing counterparts. Other autistic participants were sorted into two classes characterized by different patterns of factor loadings. In each class, these loadings corresponded to even more atypical SSP subscores on various subscales. However, while one of these classes–viz., 2-GPL–was characterized by generally atypical sensory processing, factor loadings suggested that overall SSP performance in class 1-LEW was heavily shaped by the SSP’s LEW subscale, though raw scores on other subscales were low as well. The finding that LEW subscores can so heavily influence overall SSP performance suggests that clinicians and researchers should exercise caution in interpreting the SSP and closely examine not only overall scores but also scores on each subscale. Unfortunately, the meaning of the LEW subscale is somewhat unclear; low LEW scores in ASD were weakly to moderately associated with low scores on other SSP subscales, with poor motor skills, with high internalizing, and with high cognitive abilities.

The present study also provides evidence that caregiver-reported sensory processing on the SSP converges with auditory event-related potential amplitudes in ASD. Specifically, neural hyper-reactivity to loud sounds was observed in class 2-GPL, which was characterized by low SSP scores, including high levels of sensory sensitivity in various sensory modalities such as touch and hearing. Thus, neural hyper-reactivity to loud sounds appeared to be associated not only with hyperacusis but also sensory processing in other modalities. Broadly speaking, the present study’s finding of convergence between neural and caregiver-report measures furthers progress towards understanding the relationships among different types of sensory processing measures in ASD.

Finally, the present study found increased anxiety and sleep disturbances among autistic participants in classes 1-LEW and 2-GPL, both characterized by more positive factor loadings and lower SSP subscores, relative to class 3-NL. Given the importance of anxiety and sleep in the daily experiences of individuals, this result emphasizes the importance of understanding the heterogeneous sensory phenotypes and experiences of autistic people.

## Supplementary Information

Below is the link to the electronic supplementary material.

Supplementary file1 (DOCX 783 KB)

## Footnotes

1 Given the expressed preferences of many autistic people (see Kenny et al., 2016), and in an effort to avoid embedding political value judgements into scientific and clinical terminology, we have here avoided use of terms such as “disorder” or “condition” to describe autism, opting to employ the more neutral terms “autism spectrum development” (per Dwyer et al., 2020b) and “autism.” Similarly, in deference to the preferences of many autistic people (Kenny et al.) and in light of possible links between person-first language and stigma (see Gernsbacher, 2017), we have employed identity-first language (e.g., “autistic”) in preference to person-first language (e.g., “person with autism”).

2 Note that in the present study, participants with small or unclear P1 responses were included in analyses–unlike in Dwyer et al. (2021)–in order to prevent confounding the amplitude analysis; Dwyer et al. only measured latency and they did not require complete data for ANOVA analyses.

3 In fact, Tomchek et al. appear to suggest the opposite, but their discussion seems to reverse the direction of an observed association between SSP LEW scores and language outcomes.
