# Supplementary material for: Exploring Sensory Subgroups in Typical Development and Autism Spectrum Development Using Factor Mixture Modelling
Source: J Autism Dev Disord. 2021 Sep 9;52(9):3840–60. doi: 10.1007/s10803-021-05256-6 (PMC9349169; doi:10.1007/s10803-021-05256-6)
Supplement: Supplementary file 1 — Supplementary file1 (DOCX 783 KB) [file 10803_2021_5256_MOESM1_ESM.docx]

**Supplementary Materials**

**Alternative Class Solutions**

**Two-Class Solution.**

The first class in the two-class model (i.e., class 2.1) was characterized by negative loadings of the subscales onto the latent factor (Supplementary Table 1), comparable to the third or NL class described in the main text. Meanwhile, the second class in the two-class model (i.e., class 2.2) was dominated by an extremely high loading of the low energy/weak subscale onto the latent factor, comparable to the first or LEW class described in the main text.

| Supplementary Table 1. *Estimated factor loadings, along with associated standard errors, from each class in the two-class model.* | | | | | | |
| --- | --- | --- | --- | --- | --- | --- |
|  | Class 2.1  Estimate (*SE*) | | | Class 2.2  Estimate (*SE*) | | |
|  | Raw | Standardized | | Raw | Standardized | |
|  |  | Time 1 | Time 3 |  | Time 1 | Time 3 |
| LEW | –0.638 (0.209) | –0.343 (0.079) | –0.205 (0.080) | 11.262 (0.833) | 0.988 (0.004) | 0.965 (0.016) |
| TSS | –3.357 (0.272) | –0.606 (0.042) | –0.612 (0.045) | 1.545 (0.774) | 0.331 (0.147) | 0.335 (0.149) |
| HYI | –4.183 (0.231) | –0.806 (0.024) | –0.780 (0.036) | 1.897 (0.612) | 0.525 (0.128) | 0.492 (0.122) |
| TS | –1.959 (0.183) | –0.645 (0.043) | –0.664 (0.042) | 1.559 (0.433) | 0.557 (0.109) | 0.577 (0.105) |
| MS | –0.780 (0.164) | –0.384 (0.066) | –0.399 (0.071) | 1.509 (0.329) | 0.627 (0.093) | 0.644 (0.090) |
| AD | –1.857 (0.179) | –0.718 (0.041) | –0.660 (0.053) | 1.656 (0.346) | 0.677 (0.079) | 0.616 (0.090) |
| HRS | –1.792 (0.107) | –0.773 (0.033) | –0.776 (0.033) | 0.815 (0.232) | 0.484 (0.111) | 0.488 (0.111) |
| VS | –0.920 (0.128) | –0.503 (0.054) | –0.451 (0.054) | 1.613 (0.355) | 0.714 (0.081) | 0.663 (0.085) |
| ND | –0.171 (0.143) | –0.551 (0.054) | –0.521 (0.057) | 0.896 (0.231) | 0.451 (0.095) | 0.423 (0.100) |

The second LEW-like class were overwhelmingly dominated by autistic participants, while the first NL-like class was a mixed group of typically-developing and autistic participants (Supplementary Table 2). A Fisher’s exact test indicated that the proportions of participants from each diagnostic group were not homogeneous across classes, *p* < .0001.

| Supplementary Table 2. *Count of participants in each diagnostic group and class from the two-class solution, as well as percentage of participants from each diagnostic group assigned to a given class.* | | | | |
| --- | --- | --- | --- | --- |
|  | Class 2.1 | | Class 2.2 | |
|  | ASD | TD | ASD | TD |
| Count | 138 | 93 | 52 | 2 |
| Percentage | 72.63% | 97.89% | 27.37% | 2.11% |

Notably, no apparent analogue of the GPL class from the three-class model was present in the two-class model. Of the 25 participants assigned to the GPL class in the three-class model, 23 (or 92%) were assigned to the first, NL-like class in the two-class model.

**Four-Class Solution.**

The first class in the four-class model (i.e., class 4.1) was characterized by negative loadings of the subscales onto the latent factor (Supplementary Table 3), comparable to the third or NL class described in the main text. Meanwhile, the second class in the four-class model (i.e., class 4.2) was dominated by an extremely high loading of the LEW subscale onto the latent factor, comparable to the first or LEW class described in the main text. However, class 4.4 exhibited a similarly high loading of the LEW subscale on the latent factor. The major difference between classes 4.2 and 4.4 appeared to be in the pattern of the other loadings: class 4.2 appeared to have relatively low loadings of subscales reflecting sensory sensitivity in different modalities (viz., TSS, TS, MS, VS, and ND), as well as on the auditory distractibility and hypo-responsiveness to speech subscales. In contrast, in class 4.2, the hyperactivity and inattention subscale appeared to load more strongly on the latent factor than in class 4.4. (Note that the factor loading estimate relate to the relative contributions of these variables to the latent factor; they do not necessarily imply the existence of differences between classes in raw scores from all of these subscales.) Finally, class 4.3 appeared comparable to the generalized atypical or GPL class described in the main text.

| Supplementary Table 3. *Estimated factor loadings, along with associated standard errors, from each class in the four-class model.* | | | | | | | | | | | | |
| --- | --- | --- | --- | --- | --- | --- | --- | --- | --- | --- | --- | --- |
|  | Class 4.1  Estimate (*SE*) | | | Class 4.2  Estimate (*SE*) | | | Class 4.3  Estimate (*SE*) | | | Class 4.4  Estimate (*SE*) | | |
|  | Raw | Standardized | | Raw | Standardized | | Raw | Standardized | | Raw | Standardized | |
|  |  | Time 1 | Time 3 |  | Time 1 | Time 3 |  | Time 1 | Time 3 |  | Time 1 | Time 3 |
| LEW | −0.467 (0.188) | −0.260 (0.082) | −0.164 (0.089) | 11.630 (1.359) | 0.989 (0.004) | 0.972 (0.016) | 1.379 (0.739) | 0.623 (0.213) | 0.441 (0.200) | 10.171 (1.185) | 0.986 (0.005) | 0.964 (0.020) |
| TSS | −3.055 (0.292) | −0.570 (0.046) | −0.575 (0.049) | 1.063 (1.072) | 0.235 (0.223) | 0.237 (0.229) | 4.310 (0.816) | 0.700 (0.073) | 0.704 (0.079) | 1.832 (1.011) | 0.384 (0.181) | 0.388 (0.178) |
| HYI | −4.112 (0.244) | −0.804 (0.026) | −0.774 (0.034) | 2.354 (0.816) | 0.612 (0.140) | 0.574 (0.138) | 3.892 (0.719) | 0.788 (0.064) | 0.757 (0.066) | 1.365 (0.868) | 0.409 (0.224) | 0.376 (0.201) |
| TS | −1.570 (0.177) | −0.567 (0.048) | −0.593 (0.046) | 0.681 (0.452) | 0.286 (0.174) | 0.304 (0.188) | 3.260 (0.721) | 0.820 (0.065) | 0.837 (0.062) | 2.465 (0.674) | 0.734 (0.100) | 0.756 (0.088) |
| MS | −0.114 (0.077) | −0.074 (0.050) | −0.124 (0.084) | 0.492 (0.286) | 0.306 (0.172) | 0.473 (0.213) | 4.158 (0.650) | 0.938 (0.024) | 0.977 (0.012) | 3.175 (0.378) | 0.901 (0.028) | 0.961 (0.018) |
| AD | −1.504 (0.180) | −0.640 (0.049) | −0.569 (0.060) | 1.442 (0.544) | 0.624 (0.140) | 0.553 (0.158) | 2.919 (0.526) | 0.850 (0.050) | 0.802 (0.058) | 1.894 (0.484) | 0.724 (0.099) | 0.657 (0.097) |
| HRS | −1.826 (0.111) | −0.791 (0.031) | −0.776 (0.031) | 0.522 (0.387) | 0.346 (0.229) | 0.331 (0.221) | 1.449 (0.421) | 0.716 (0.111) | 0.698 (0.103) | 0.898 (0.250) | 0.536 (0.111) | 0.517 (0.110) |
| VS | −0.746 (0.155) | −0.429 (0.072) | −0.386 (0.071) | 0.858 (0.574) | 0.480 (0.239) | 0.433 (0.234) | 1.317 (0.537) | 0.643 (0.162) | 0.594 (0.157) | 2.032 (0.499) | 0.792 (0.082) | 0.751 (0.085) |
| ND | −0.966 (0.165) | −0.480 (0.063) | −0.447 (0.072) | 0.693 (0.333) | 0.366 (0.154) | 0.338 (0.150) | 1.909 (0.469) | 0.734 (0.090) | 0.703 (0.092) | 1.065 (0.325) | 0.517 (0.122) | 0.483 (0.116) |

Classes 4.2 through 4.4 were overwhelmingly dominated by autistic participants, while class 4.1 included a mixture of autistic and typically-developing individuals. A Fisher’s exact test indicated that the proportions of participants from each diagnostic group were not homogeneous across classes, *p* < .0001.

| Supplementary Table 4. *Count of participants in each diagnostic group and class from the four-class solution, as well as percentage of participants from each diagnostic group assigned to a given class.* | | | | | | | | |
| --- | --- | --- | --- | --- | --- | --- | --- | --- |
|  | Class 4.1 | | Class 4.2 | | Class 4.3 | | Class 4.4 | |
|  | ASD | TD | ASD | TD | ASD | TD | ASD | TD |
| Count | 115 | 90 | 22 | 1 | 19 | 3 | 34 | 1 |
| Percentage | 60.53% | 94.74% | 11.58% | 1.05% | 10.00% | 3.16% | 17.89% | 1.05% |

All four classes were tightly related to classes in the three-class solution. All 23 participants in class 4.2 were assigned to the LEW class in the three-class model, as were 31 of 35 (or 88.57% of) participants in class 4.4. Meanwhile, 20 of 22 (or 90.91% of) participants in class 4.3 were assigned to the GPL class in the three-class model, while 202 of the 205 (or 98.54% of) participants in class 4.1 were assigned to the NL class in the three-class model.

**Exploration of LEW Scores**

Given the dominant role of LEW subscores in shaping overall SSP performance in class 1-LEW, additional analyses were conducted in order to better understand the LEW subscale. Specifically, Pearson correlation coefficients between raw LEW subscores and other variables – namely, MSEL fine motor and visual reception T-scores, VABS fine and gross motor V-scores, CSHQ sleep disturbance scores, CBCL externalizing and internalizing T-scores, and SSP subscores were explored within the ASD group at Time 1 (Supplementary Table 5) at Time 3 (Supplementary Table 6). Partial correlations were also explored, controlling for age and MSEL VDQ (at Time 1) and age and DAS GCA (at Time 3); note that MSEL VDQ rather than DQ was used as a covariate at Time 1 due to the potential relevance of the nonverbal MSEL fine motor subscale to LEW scores.

All Cook’s distances from significant associations were < .375 at Time 1 and < .25 at Time 3. Removal of the most outlying data point from each association did reduce observed associations with MSEL fine motor T-scores, SSP tactile sensitivity, and SSP auditory distractibility below significance threshold at Time 1; similarly, outlier removal reduced associations with SSP visual sensitivity and SSP noise distress below significance threshold at Time 3.

It may be worth noting that MSEL and VABS fine motor scores show opposite patterns of correlation with LEW scores. This seemingly anomalous finding may be explained by the MSEL fine motor scores’ covariance with other MSEL scales, as controlling for MSEL VDQ eliminated any relation between MSEL fine motor T-scores and SSP LEW raw scores. In contrast, VABS fine and gross motor scores might tap less into overall cognitive ability and more into everyday motor difficulties.

| Supplementary Table 5. *Pearson’s correlation coefficients measuring linear associations between SSP LEW scores and other variables at Time 1 in the ASD group, along with partial Pearson’s correlations controlling for chronological age and MSEL verbal developmental quotient.*^1^ | | | |
| --- | --- | --- | --- |
|  | Raw Correlation | Partial Correlation (controlling for Age) | Partial Correlation (controlling for Age, MSEL VDQ) |
| Age | −.09 |  |  |
| MSEL VDQ | −.22** | −.22** |  |
| MSEL Fine Motor T | −.18* | −.19* | −.03 |
| MSEL Visual Reception T | −.24** | −.24** | −.09 |
| VABS Fine Motor V | .19* | .18* | .29*** |
| VABS Gross Motor V | .34*** | .33*** | .35*** |
| CSHQ Total | −.19* | −.19* | −.18* |
| CBCL Externalizing T | .03 | .03 | .03 |
| CBCL Internalizing T | −.22** | −.23** | −.22** |
| SSP TSS | .02 | .02 | .03 |
| SSP HYI | .09 | .10 | .13 |
| SSP TS | .18* | .18* | .17* |
| SSP MS | .31*** | .32*** | .28*** |
| SSP AD | .16* | .15 | .13 |
| SSP HRS | −.06 | −.05 | −.02 |
| SSP VS | .37*** | .37*** | .35*** |
| SSP ND | .10 | .09 | .07 |
| ^1^ Asterisk (*) indicates that the *p* corresponding to the Pearson’s correlation coefficient is < .05, double (**) indicates *p* < .01, and triple (***) indicates *p* < .001, uncorrected. | | | |

| Supplementary Table 6. *Pearson’s correlation coefficients measuring linear associations between SSP LEW scores and other variables at Time 3 in the ASD group, along with partial Pearson’s correlations controlling for chronological age and DAS GCA.*^1^ | | | |
| --- | --- | --- | --- |
|  | Raw Correlation | Partial Correlation (controlling for Age) | Partial Correlation (controlling for Age, DAS GCA) |
| Age | −.32** |  |  |
| DAS GCA | −.18 | −.22 |  |
| VABS Fine Motor V | .19 | .15 | .37** |
| VABS Gross Motor V | .45*** | .43*** | .44*** |
| CSHQ Total | −.15 | −.14 | −.09 |
| CBCL Externalizing T | −.12 | −.04 | −.01 |
| CBCL Internalizing T | −.38*** | −.31** | −.34** |
| SSP TSS | .03 | .09 | .13 |
| SSP HYI | .02 | −.01 | −.04 |
| SSP TS | .30** | .34** | .31** |
| SSP MS | .41*** | .39*** | .42*** |
| SSP AD | .41*** | .41*** | .40*** |
| SSP HRS | .24* | .21* | .14 |
| SSP VS | .24* | .23* | .31** |
| SSP ND | .23* | .23* | .26* |
| ^1^ Asterisk (*) indicates that the *p* corresponding to the Pearson’s correlation coefficient is < .05, double (**) indicates *p* < .01, and triple (***) indicates *p* < .001, uncorrected. | | | |

**SSP Scores Across Time Points**

Paired Wilcoxon signed-rank tests were used to compare SSP scores across time points 1 and 3 in each group and class (Supplementary Tables 7-10). Cliff’s δ was used as an effect size metric, with a 95% paired-samples confidence interval being calculated based on the of overall movement of the ordinal distribution (the “between” column in output from R’s orddom package; Rogmann, 2013). For the most part, sensory scores were stable over time. However, autistic participants in class 1-LEW were also reported to have greater auditory distractibility (i.e., lower AD scores) at Time 3 than Time 1, which might be consistent with the reported change in noise distress if auditory distractibility is interpreted to reflect general problems functioning amid auditory stimulation (rather than, for example, characteristics of ADHD); notably, the auditory distractibility effect was large enough that it could have survived a correction for multiple comparisons.

In the typically-developing group, participants in class 3-NL (that is, the vast majority of participants) were reportedly less hyperactive/inattentive at Time 3 than at Time 1. This effect of hyperactivity/inattentiveness was large enough that it would be able to survive correction for the thirty-six comparisons included in this analysis.

| Supplementary Table 7. *SSP subscores of autistic participants in class 1-LEW at Time 1 and Time 3. Means and standard deviations are given both for all participants and specifically for those who have both Time 1 and Time 3 data, the latter of whom are included in paired Wilcoxon signed-rank statistical tests comparing Time 1 and Time 3 subscores.* | | | | | | | |
| --- | --- | --- | --- | --- | --- | --- | --- |
|  | All ASD Participants | | ASD Participants with Both Timepoints | | | | |
|  | Mean (SD) | | Mean (SD) | | *p* | Cliff’s δ | |
|  | T1 | T3 | T1 | T3 |  | Value | 95% CI |
| LEW | 18.90 (6.45) | 16.61 (5.99) | 20.08 (6.97) | 17.04 (5.82) | .10 | .27 | −.02, .52 |
| TSS | 12.52 (5.07) | 12.47 (4.88) | 12.85 (4.97) | 12.50 (5.19) | >.99 | .04 | −.20, .29 |
| HYI | 13.67 (3.65) | 14.81 (4.13) | 13.77 (4.18) | 14.81 (4.50) | .44 | −.13 | −.39, .15 |
| TS | 15.57 (3.22) | 16.45 (2.38) | 15.40 (3.70) | 16.60 (2.52) | .22 | −.17 | −.42, .11 |
| MS | 12.38 (1.85) | 12.19 (2.51) | 12.62 (1.72) | 12.23 (2.61) | .56 | .03 | −.27, .32 |
| AD | 11.32 (2.12) | 9.32 (2.63) | 11.54 (2.18) | 9.58 (2.67) | .0009 | .44 | .18, .64 |
| HRS | 4.92 (1.37) | 5.45 (1.67) | 4.73 (1.37) | 5.35 (1.70) | .14 | −.28 | −.51, −.01 |
| VS | 6.89 (2.42) | 7.52 (2.20) | 8.04 (2.05) | 7.58 (2.25) | .09 | .15 | −.07, .36 |
| ND | 7.46 (1.54) | 6.06 (2.14) | 7.54 (1.48) | 6.08 (2.28) | .01 | .40 | .09, .64 |

| Supplementary Table 8. *SSP subscores of autistic participants in class 2-GPL at Time 1 and Time 3. Means and standard deviations are given both for all participants and specifically for those who have both Time 1 and Time 3 data, the latter of whom are included in paired Wilcoxon signed-rank statistical tests comparing Time 1 and Time 3 subscores.* | | | | | | | |
| --- | --- | --- | --- | --- | --- | --- | --- |
|  | All ASD Participants | | ASD Participants with Both Timepoints | | | | |
|  | Mean (SD) | | Mean (SD) | | *p* | Cliff’s δ | |
|  | T1 | T3 | T1 | T3 |  | Value | 95% CI |
| LEW | 27.05 (3.32) | 23.79 (6.03) | 27.60 (3.66) | 22.50 (6.47) | .04 | .46 | −.03, .77 |
| TSS | 9.85 (5.71) | 10.79 (6.02) | 12.80 (6.12) | 12.10 (6.61) | .83 | .11 | −.30, .49 |
| HYI | 11.90 (3.19) | 9.71 (2.64) | 11.60 (4.03) | 10.40 (2.59) | .32 | .09 | −.24, .40 |
| TS | 12.30 (3.42) | 13.79 (3.66) | 13.20 (3.12) | 13.60 (3.86) | .92 | −.02 | −.49, .47 |
| MS | 9.85 (3.15) | 10.23 (2.24) | 10.22 (2.91) | 10.44 (2.51) | .95 | −.06 | −.68, .62 |
| AD | 9.30 (3.44) | 8.64 (1.69) | 10.20 (3.79) | 8.60 (1.78) | .23 | .23 | −.33, .68 |
| HRS | 3.75 (1.62) | 5.00 (1.66) | 4.40 (1.65) | 4.90 (1.73) | .55 | −.23 | −.63, .25 |
| VS | 6.15 (2.43) | 7.07 (2.34) | 6.50 (2.68) | 6.80 (2.20) | .68 | −.07 | −.52, .42 |
| ND | 5.15 (2.43) | 6.07 (2.34) | 5.80 (2.57) | 6.40 (1.96) | .61 | −.23 | −.78, .51 |

| Supplementary Table 9. *SSP subscores of autistic participants in class 3-NL at Time 1 and Time 3. Means and standard deviations are given both for all participants and specifically for those who have both Time 1 and Time 3 data, the latter of whom are included in paired Wilcoxon signed-rank statistical tests comparing Time 1 and Time 3 subscores.* | | | | | | | |
| --- | --- | --- | --- | --- | --- | --- | --- |
|  | All ASD Participants | | ASD Participants with Both Timepoints | | | | |
|  | Mean (SD) | | Mean (SD) | | *p* | Cliff’s δ | |
|  | T1 | T3 | T1 | T3 |  | Value | 95% CI |
| LEW | 28.83 (1.84) | 28.14 (2.83) | 28.06 (2.51) | 27.88 (2.86) | .89 | .01 | −.17, .18 |
| TSS | 12.65 (5.57) | 12.56 (5.42) | 14.16 (5.74) | 12.53 (5.54) | .18 | .18 | −.01, .36 |
| HYI | 14.45 (4.59) | 14.76 (4.79) | 14.61 (5.32) | 14.36 (4.46) | .70 | .00 | −.20, .21 |
| TS | 16.90 (2.65) | 17.63 (2.22) | 16.81 (2.64) | 17.65 (2.67) | .08 | −.17 | −.41, .09 |
| MS | 14.23 (1.26) | 14.61 (0.77) | 14.06 (1.63) | 14.56 (0.80) | .05* | −.17 | −.35, .02 |
| AD | 12.05 (2.40) | 11.17 (2.65) | 12.09 (2.49) | 11.09 (2.80) | .15 | .22 | −.03, .44 |
| HRS | 4.90 (1.67) | 5.95 (1.77) | 4.79 (1.92) | 5.88 (1.85) | .008 | −.37 | −.59, −.11 |
| VS | 8.31 (1.70) | 8.39 (1.96) | 8.66 (1.62) | 8.34 (1.98) | .53 | .07 | −.16, .30 |
| ND | 7.95 (2.14) | 6.60 (2.29) | 7.97 (2.17) | 6.73 (2.21) | .01 | .34 | .09, .54 |
| ^*^ For *p*-values rounded to .05, asterisk indicates that *p* < .05. | | | | | | | |

| Supplementary Table 10. *SSP subscores of typically-developing participants in class 3-NL at Time 1 and Time 3. Means and standard deviations are given both for all participants and specifically for those who have both Time 1 and Time 3 data, the latter of whom are included in paired Wilcoxon signed-rank statistical tests comparing Time 1 and Time 3 subscores.* | | | | | | | |
| --- | --- | --- | --- | --- | --- | --- | --- |
|  | All TD Participants | | TD participants with Both Timepoints | | | | |
|  | Mean (SD) | | Mean (SD) | | *p* | Cliff’s δ | |
|  | T1 | T3 | T1 | T3 |  | Value | 95% CI |
| LEW | 29.75 (0.92) | 29.43 (1.09) | 29.63 (1.18) | 29.41 (1.05) | .16 | .15 | −.03, .27 |
| TSS | 17.95 (2.70) | 18.15 (3.24) | 17.89 (3.18) | 18.13 (3.26) | .81 | −.06 | −.21, .08 |
| HYI | 19.70 (3.19) | 21.59 (3.23) | 19.80 (2.67) | 21.54 (3.40) | .0007 | −.35 | −.51, −.16 |
| TS | 18.81 (1.65) | 19.31 (1.36) | 18.85 (1.35) | 19.33 (1.30) | .009 | −.24 | −.40, −.07 |
| MS | 14.13 (1.45) | 14.44 (1.11) | 14.17 (1.10) | 14.43 (1.17) | .13 | −.17 | −.33, .00 |
| AD | 13.93 (1.17) | 13.69 (1.70) | 13.80 (1.17) | 13.67 (1.80) | .97 | −.07 | −.26, .12 |
| HRS | 8.24 (1.49) | 8.28 (1.45) | 8.07 (1.39) | 8.22 (1.49) | .59 | −.07 | −.25, .12 |
| VS | 8.92 (1.17) | 9.04 (1.50) | 8.91 (1.31) | 9.00 (1.59) | .55 | −.08 | −.24, .08 |
| ND | 8.21 (1.65) | 8.52 (1.83) | 8.15 (1.70) | 8.46 (1.94) | .23 | −.14 | −.34, .06 |

**Supplementary Descriptive Statistics**

| Supplementary Table 11. *Means and standard deviations of chronological age, MSEL DQ scores, VABS composite scores, CBCL DSM-oriented anxiety T-scores, and CSHQ total sleep disturbance scores at Time 1.* | | | | | | | | |
| --- | --- | --- | --- | --- | --- | --- | --- | --- |
|  | ASD Group | | | | | | TD Group | |
|  | Class 1 – LEW | | Class 2 – GPL | | Class 3 – NL | | Class 3 – NL | |
|  | *M* | *SD* | *M* | *SD* | *M* | *SD* | *M* | *SD* |
| Chronological Age (months) | 37.91 | 6.41 | 36.84 | 5.40 | 37.65 | 6.36 | 36.73 | 7.44 |
| MSEL DQ | 69.26 | 21.83 | 62.82 | 20.95 | 60.42 | 19.12 | 105.61 | 11.91 |
| VABS Composite | 75.44 | 12.82 | 73.82 | 8.16 | 76.28 | 11.69 | 110.71 | 11.66 |
| CBCL Anxiety T-score | 58.20 | 9.90 | 59.22 | 10.85 | 54.44 | 7.61 | 51.31 | 3.07 |
| CSHQ Total Sleep Disturbances | 46.23 | 8.39 | 51.00 | 8.21 | 42.52 | 6.95 | 41.30 | 5.99 |

| Supplementary Table 12. *Means and standard deviations of chronological age, DAS GCA scores, VABS composite scores, CBCL DSM-oriented anxiety T-scores, and CSHQ total sleep disturbance scores at Time 3.* | | | | | | | | |
| --- | --- | --- | --- | --- | --- | --- | --- | --- |
|  | ASD Group | | | | | | TD Group | |
|  | Class 1 – LEW | | Class 2 – GPL | | Class 3 – NL | | Class 3 – NL | |
|  | *M* | *SD* | *M* | *SD* | *M* | *SD* | *M* | *SD* |
| Chronological Age (months) | 73.04 | 13.95 | 69.54 | 13.89 | 65.37 | 8.27 | 67.58 | 13.77 |
| DAS GCA | 92.04 | 25.33 | 94.00 | 26.10 | 88.28 | 23.13 | 113.10 | 10.44 |
| VABS Composite | 76.45 | 13.28 | 80.73 | 18.80 | 78.81 | 16.03 | 112.71 | 11.52 |
| CBCL Anxiety T-score | 57.55 | 10.06 | 58.20 | 7.37 | 54.16 | 7.33 | 52.34 | 4.90 |
| CSHQ Total Sleep Disturbances | 42.37 | 6.98 | 45.31 | 8.89 | 41.00 | 6.42 | 38.16 | 5.78 |

| Supplementary Table 13. *Means and standard deviations of P1 ERP latencies among autistic participants at Time 1.* | | | | | | | |
| --- | --- | --- | --- | --- | --- | --- | --- |
| Intensity | Hemisphere | ASD Group | | | | | |
|  |  | Class 1 – LEW | | Class 2 – GPL | | Class 3 – NL | |
|  |  | *M* | *SD* | *M* | *SD* | *M* | *SD* |
| 50 dB | Left | 124.45 | 12.85 | 124.18 | 11.87 | 123.30 | 7.98 |
|  | Right | 122.42 | 8.74 | 124.06 | 8.79 | 122.69 | 11.46 |
| 60 dB | Left | 113.61 | 9.58 | 110.94 | 12.44 | 111.94 | 9.89 |
|  | Right | 111.03 | 12.27 | 115.24 | 10.63 | 112.67 | 10.70 |
| 70 dB | Left | 100.06 | 8.70 | 107.06 | 8.00 | 98.90 | 9.72 |
|  | Right | 101.10 | 8.84 | 98.59 | 10.86 | 100.06 | 11.03 |
| 80 dB | Left | 101.13 | 8.64 | 99.82 | 5.87 | 98.46 | 8.31 |
|  | Right | 97.03 | 7.90 | 99.00 | 8.65 | 97.45 | 9.28 |

**Supplementary Figures**

| 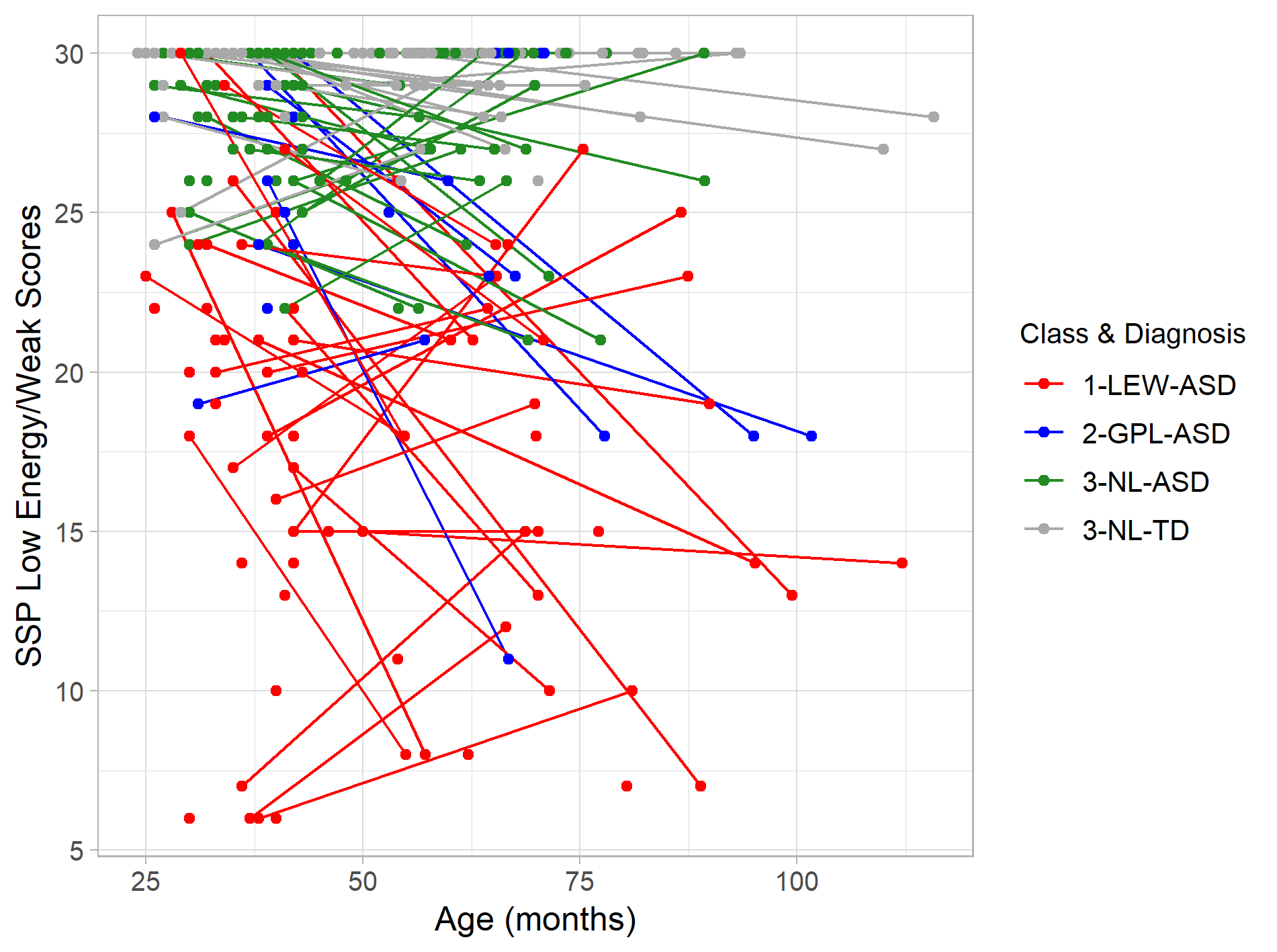  *Supplementary Figure 1*. Spaghetti plot depicting raw low energy/weakness (LEW) scores and trajectories of participants from each class and diagnosis across both time-points. Only typically-developing participants in classes 1-LEW and 2-GPL are excluded, due to small numbers. |
| --- |

| 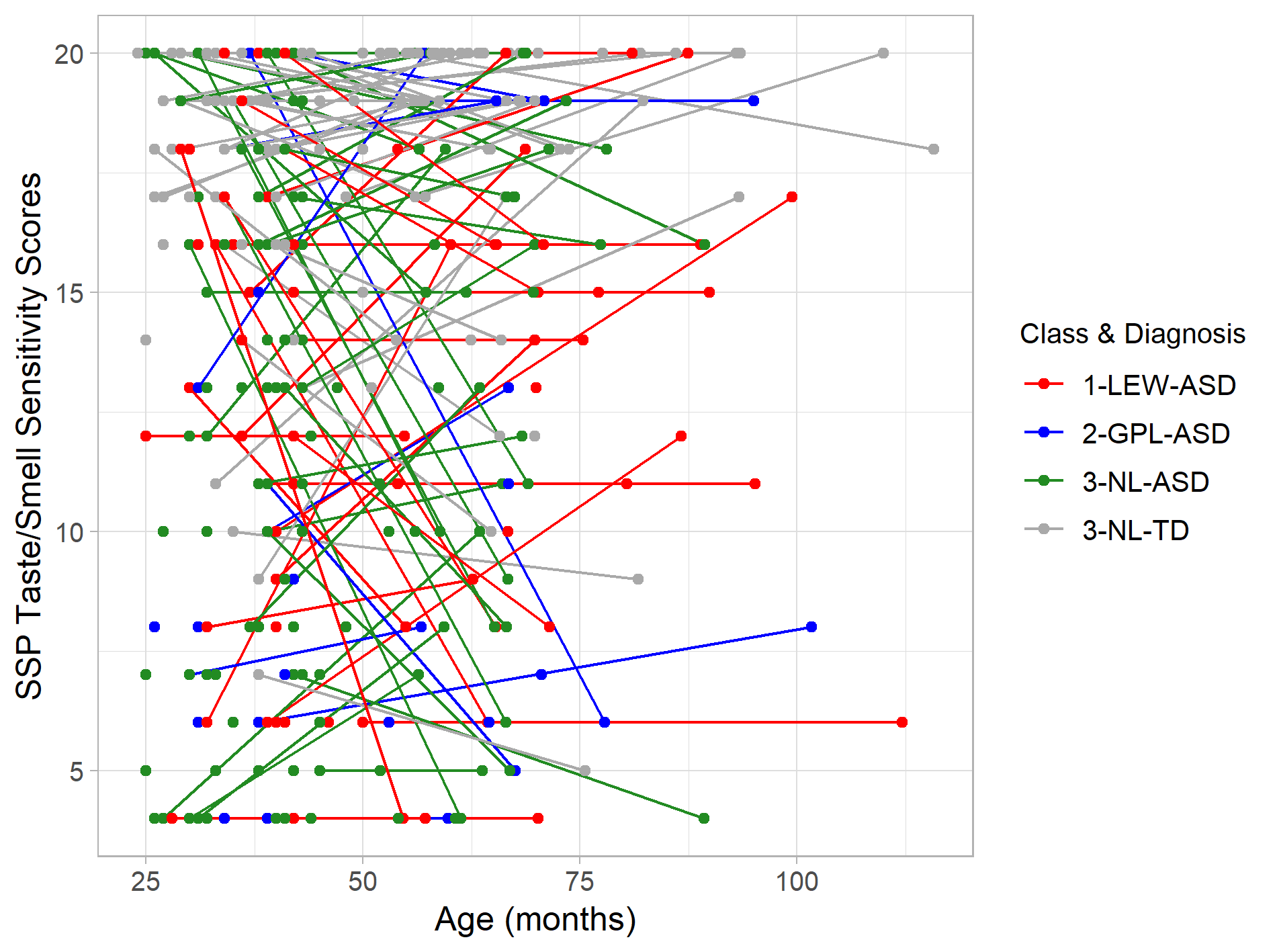  *Supplementary Figure 2*. Spaghetti plot depicting raw taste/smell sensitivity (TSS) scores and trajectories of participants from each class and diagnosis across both time-points. Only typically-developing participants in classes 1-LEW and 2-GPL are excluded, due to small numbers. |
| --- |

| 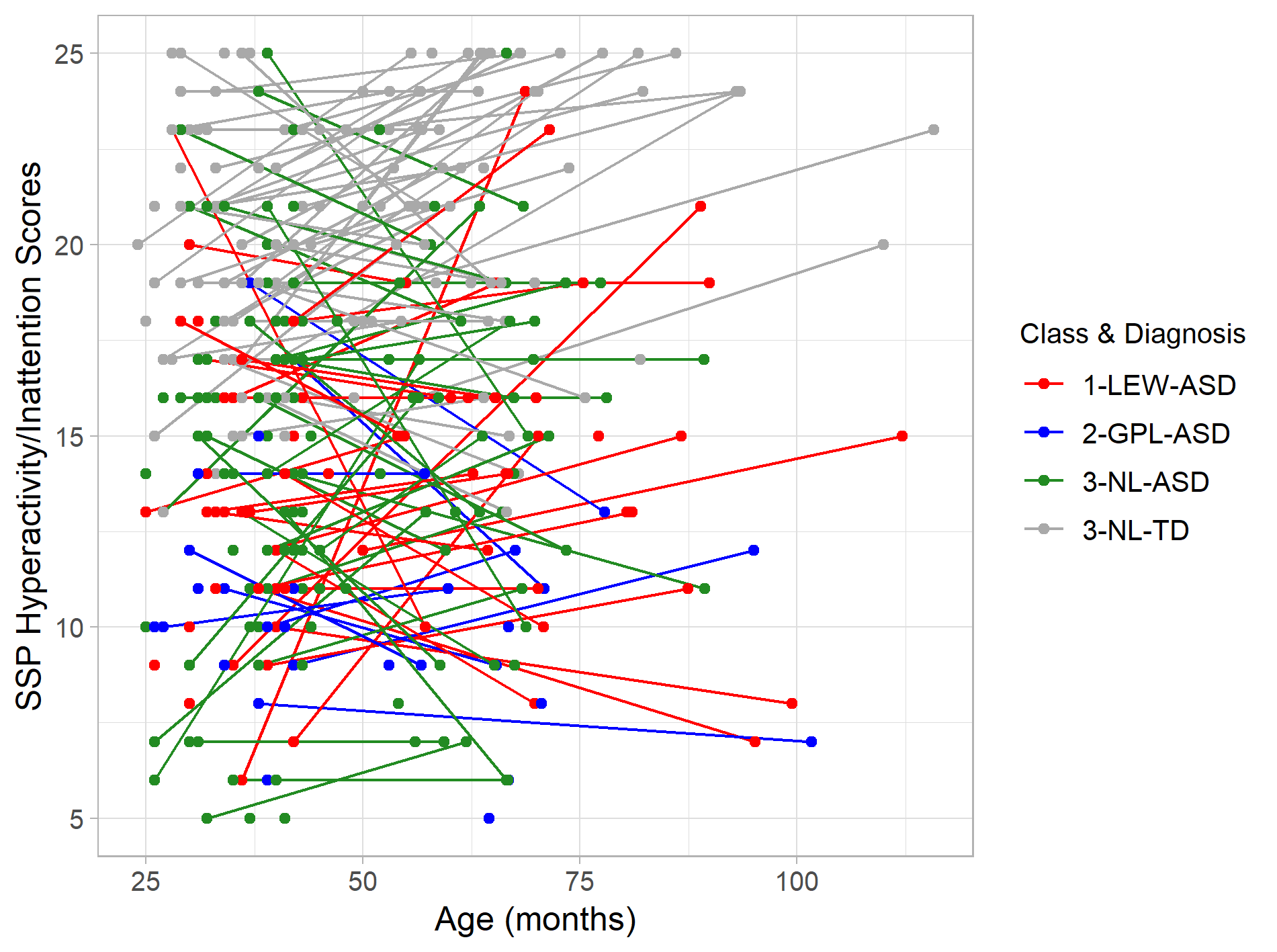  *Supplementary Figure 3*. Spaghetti plot depicting raw hyperactivity/inattention (HYI) scores and trajectories of participants from each class and diagnosis across both time-points. Only typically-developing participants in classes 1-LEW and 2-GPL are excluded, due to small numbers. |
| --- |

| 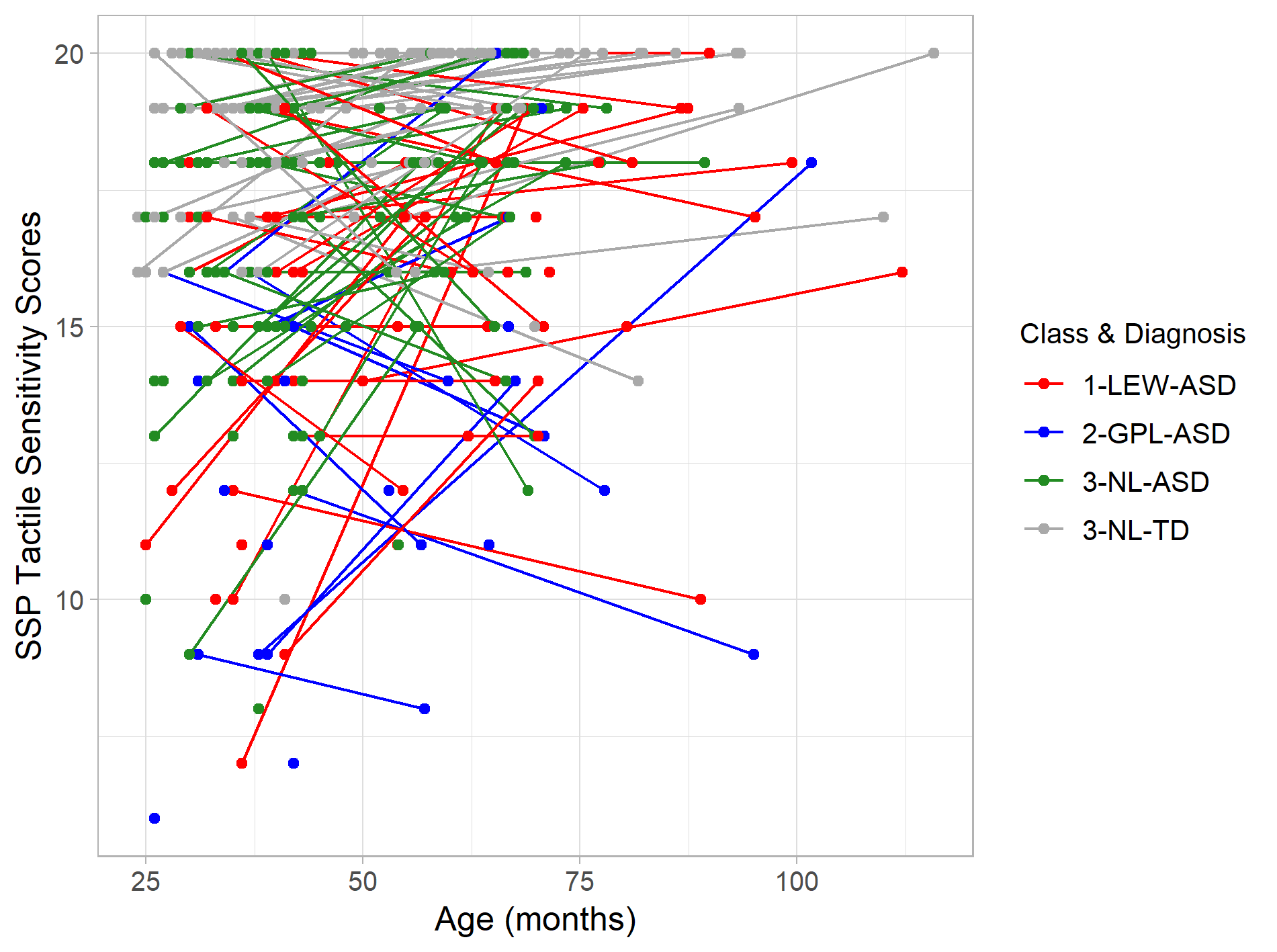  *Supplementary Figure 4*. Spaghetti plot depicting raw tactile sensitivity (TS) scores and trajectories of participants from each class and diagnosis across both time-points. Only typically-developing participants in classes 1-LEW and 2-GPL are excluded, due to small numbers. |
| --- |

| 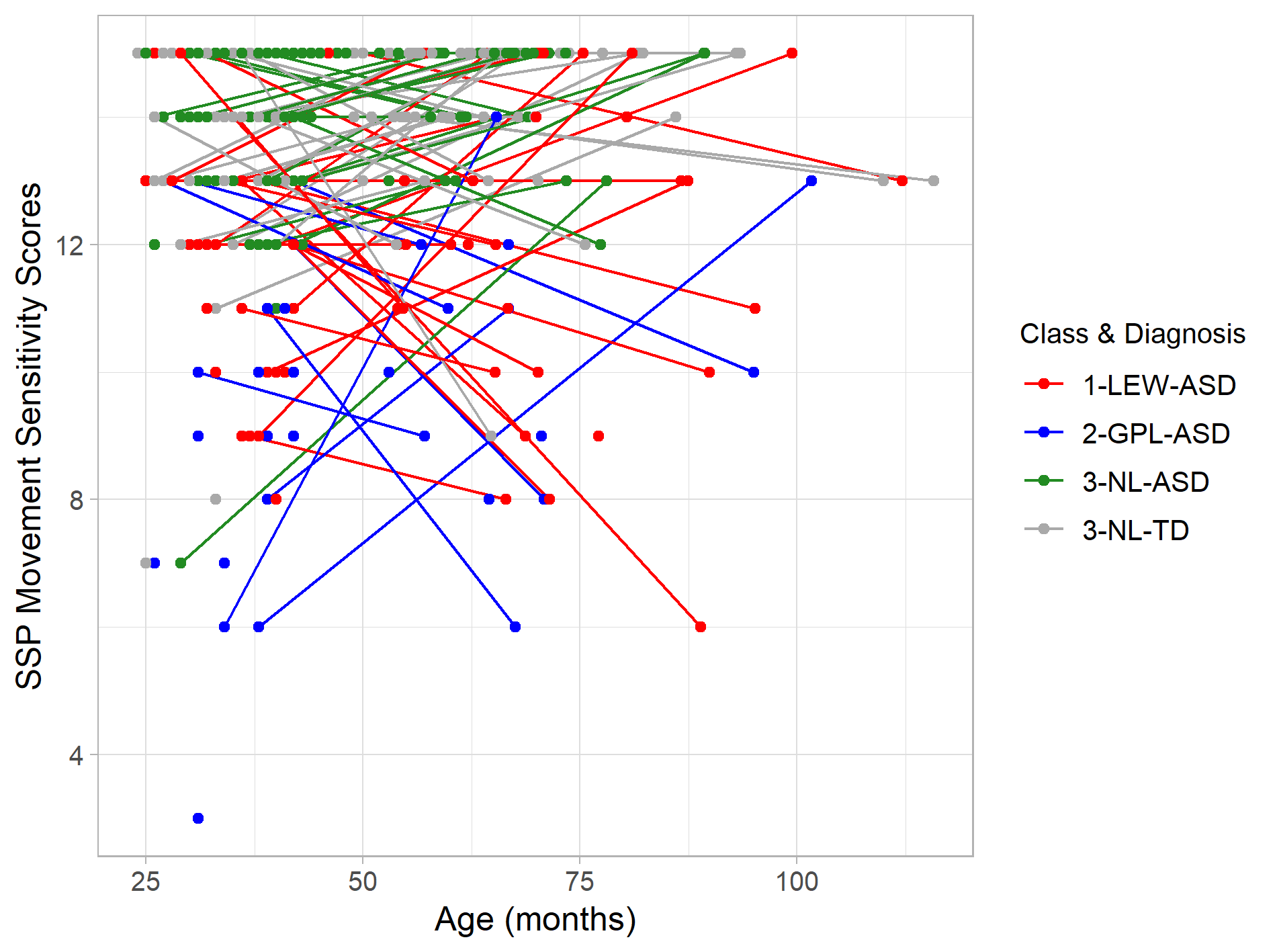  *Supplementary Figure 5*. Spaghetti plot depicting raw movement sensitivity (MS) scores and trajectories of participants from each class and diagnosis across both time-points. Only typically-developing participants in classes 1-LEW and 2-GPL are excluded, due to small numbers. |
| --- |

| 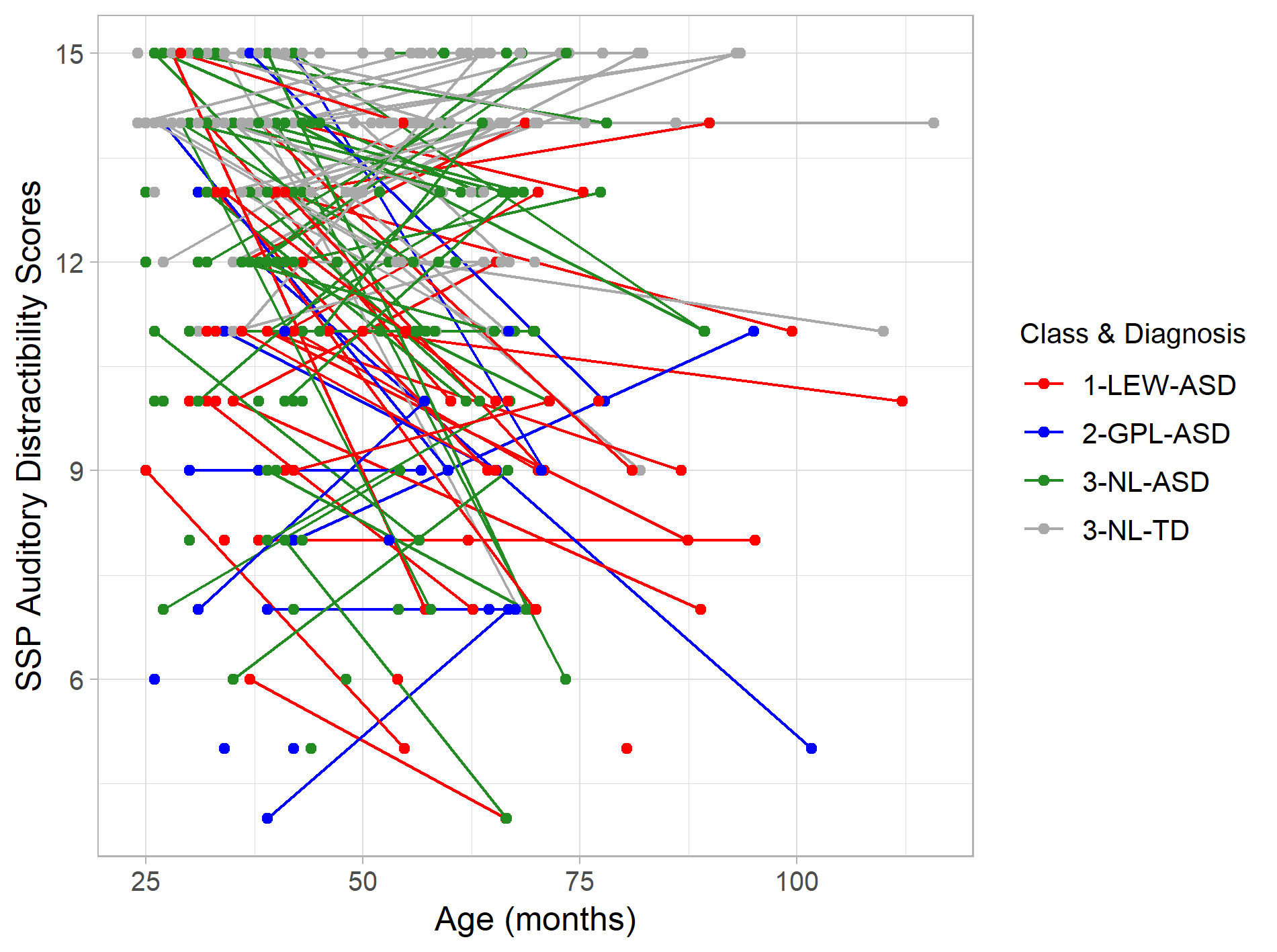  *Supplementary Figure 6*. Spaghetti plot depicting raw auditory distractibility (AD) scores and trajectories of participants from each class and diagnosis across both time-points. Only typically-developing participants in classes 1-LEW and 2-GPL are excluded, due to small numbers. |
| --- |

| 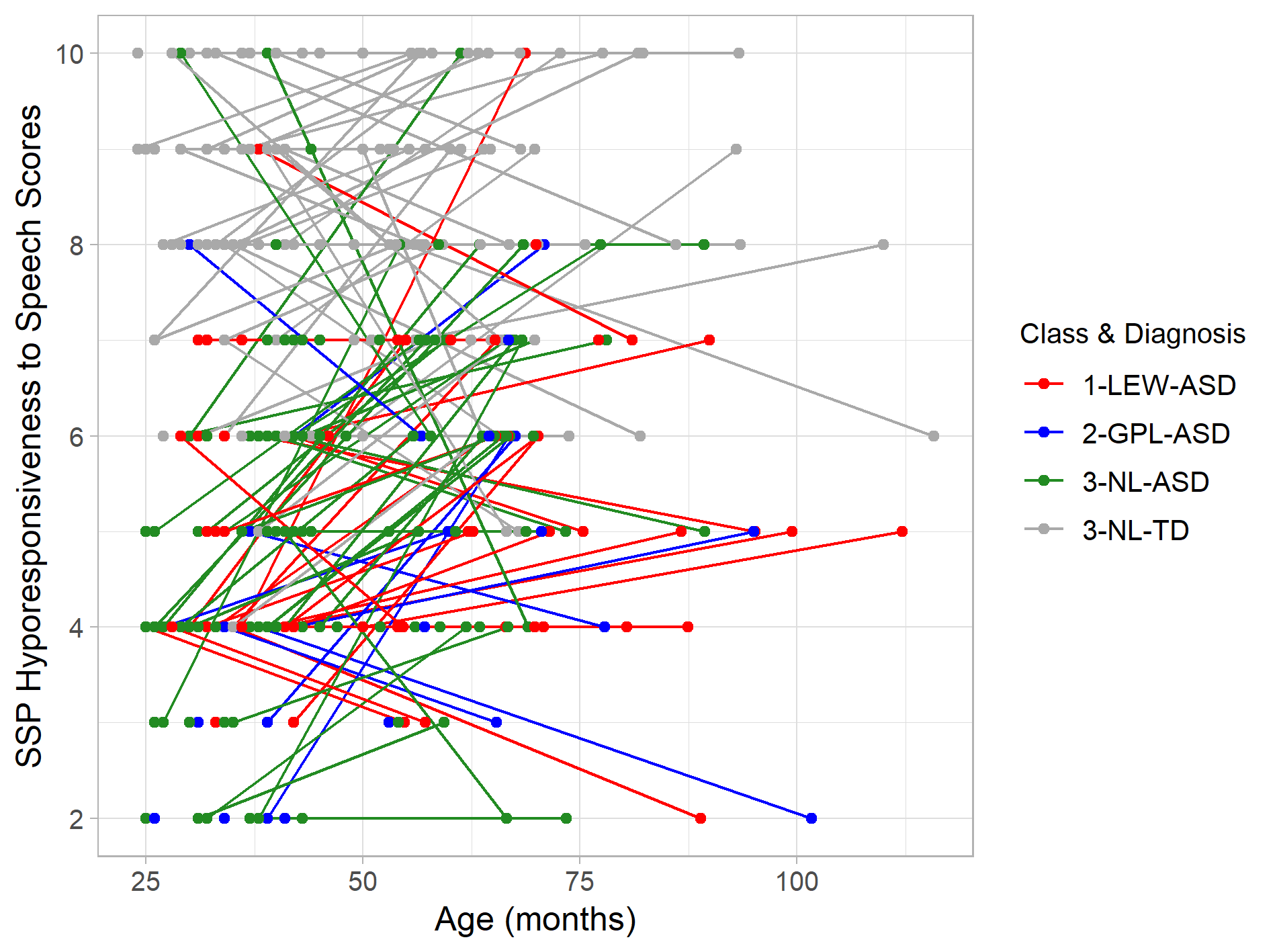  *Supplementary Figure 7*. Spaghetti plot depicting raw hypo-responsiveness to speech (HRS) scores and trajectories of participants from each class and diagnosis across both time-points. Only typically-developing participants in classes 1-LEW and 2-GPL are excluded, due to small numbers. |
| --- |

| 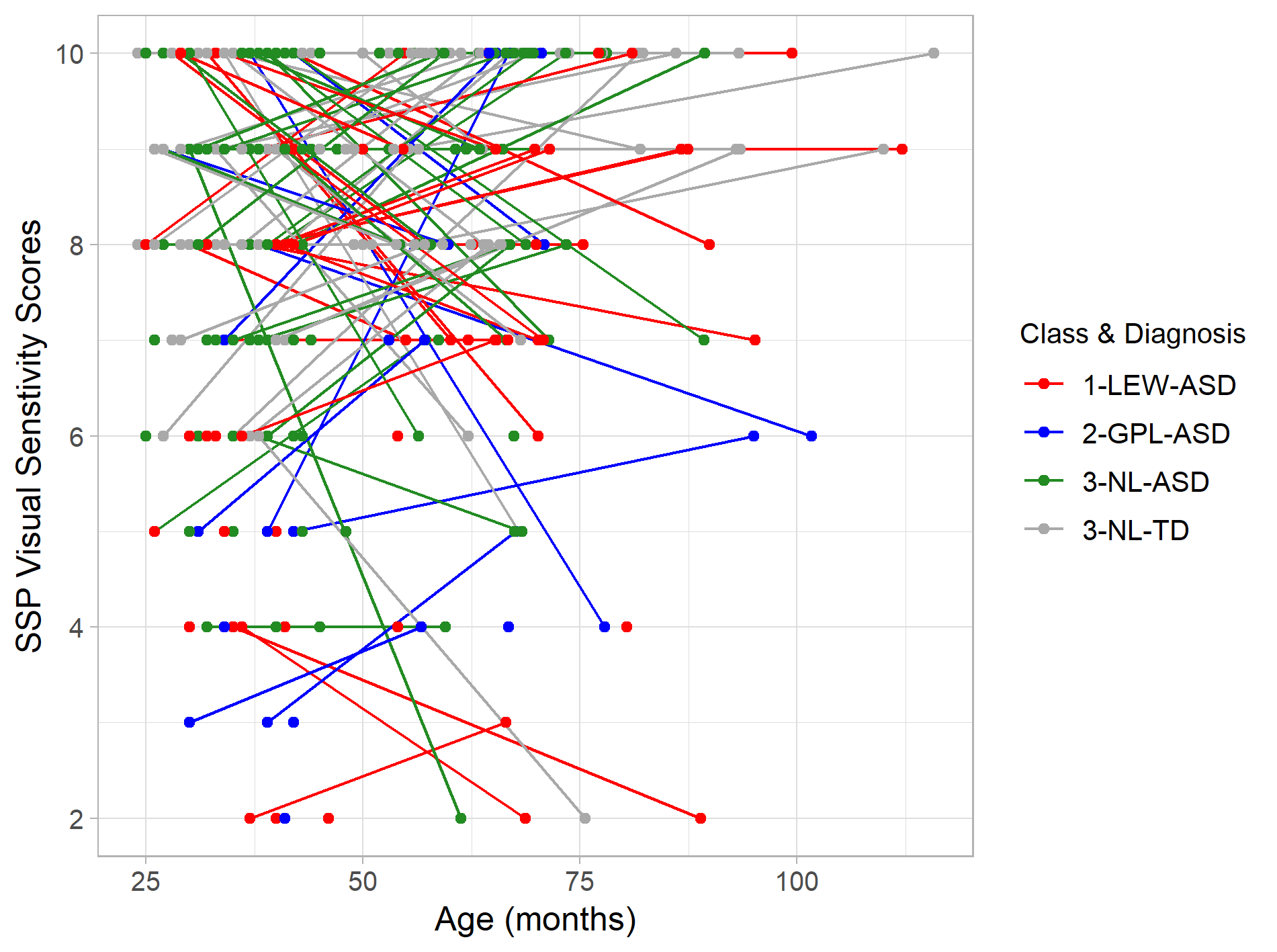  *Supplementary Figure 8*. Spaghetti plot depicting raw visual sensitivity (VS) scores and trajectories of participants from each class and diagnosis across both time-points. Only typically-developing participants in classes 1-LEW and 2-GPL are excluded, due to small numbers. |
| --- |

| 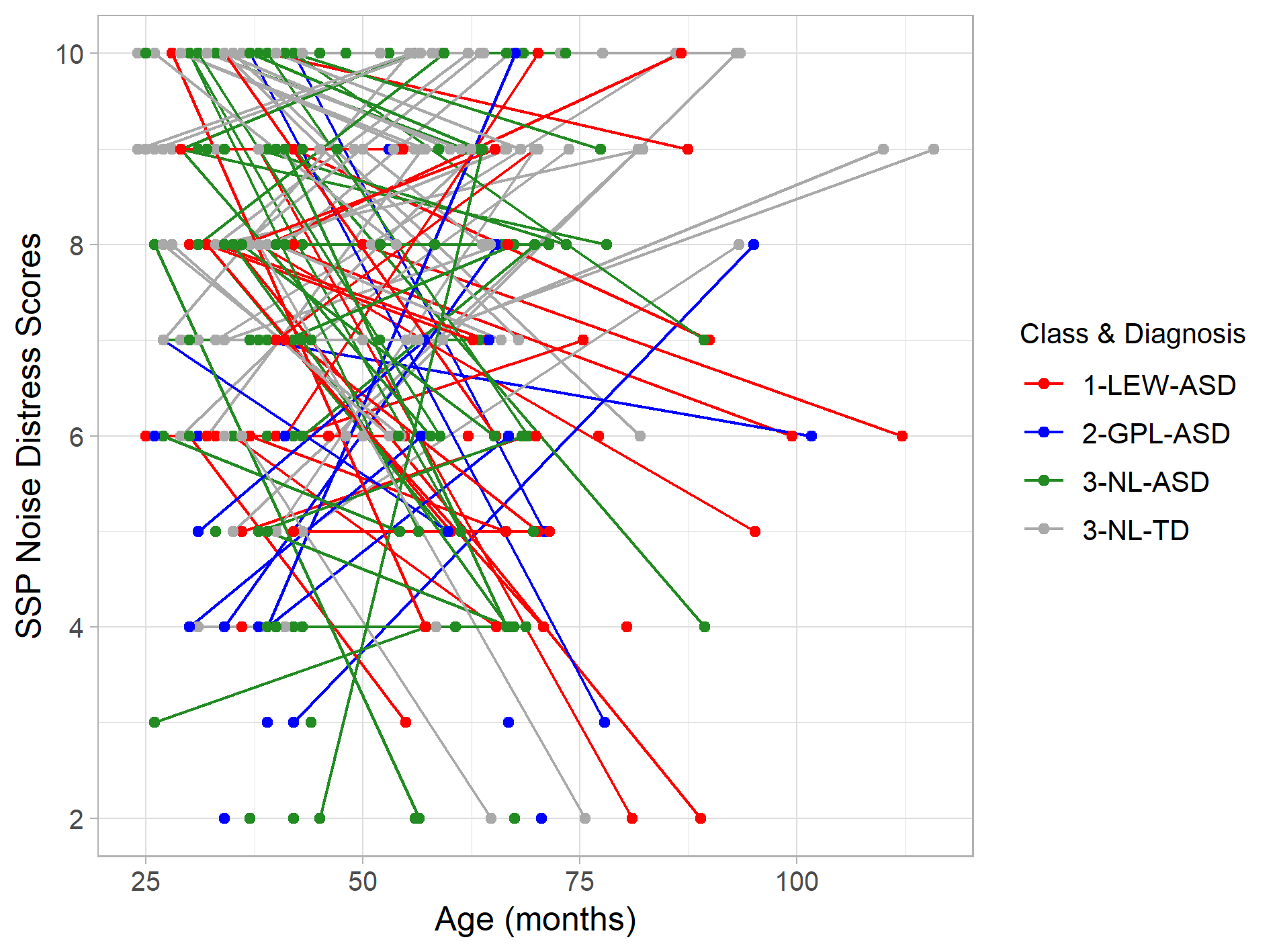  *Supplementary Figure 9*. Spaghetti plot depicting raw noise distress (ND) scores and trajectories of participants from each class and diagnosis across both time-points. Only typically-developing participants in classes 1-LEW and 2-GPL are excluded, due to small numbers. |
| --- |

| 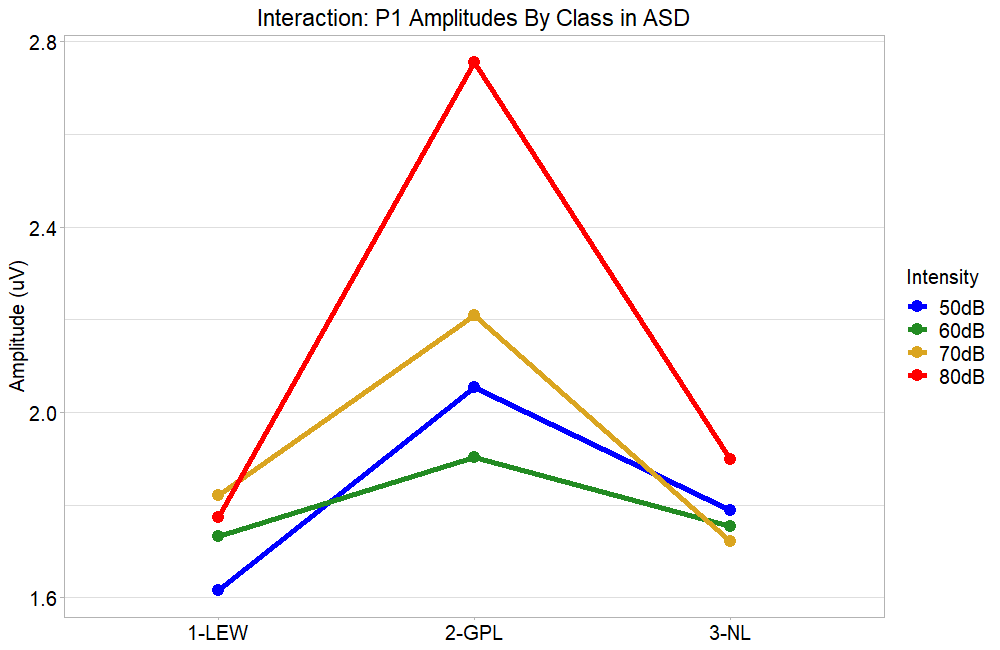  *Supplementary Figure 10*. Simple interaction plot showing mean P1 ERP amplitudes in each stimulus intensity (loudness) condition and class, collapsing across hemisphere. The 80 dB response amplitudes in class 2-GPL appear to be larger from visual inspection and this conclusion is supported by statistical tests. However, although visual inspection seems to hint at amplitude differences between 2-GPL and other classes in the other three intensity conditions, these effects did not approach statistical significance after correction for multiple comparisons. |
| --- |
